# A Study of Novel Exploratory Tools, Digital Technologies, and Central Nervous System Biomarkers to Characterize Unipolar Depression

**DOI:** 10.3389/fpsyt.2021.640741

**Published:** 2021-05-06

**Authors:** Oleksandr Sverdlov, Jelena Curcic, Kristin Hannesdottir, Liangke Gou, Valeria De Luca, Francesco Ambrosetti, Bingsong Zhang, Jens Praestgaard, Vanessa Vallejo, Andrew Dolman, Baltazar Gomez-Mancilla, Konstantinos Biliouris, Mark Deurinck, Francesca Cormack, John J. Anderson, Nicholas T. Bott, Ziv Peremen, Gil Issachar, Offir Laufer, Dale Joachim, Raj R. Jagesar, Niels Jongs, Martien J. Kas, Ahnjili Zhuparris, Rob Zuiker, Kasper Recourt, Zoë Zuilhof, Jang-Ho Cha, Gabriel E. Jacobs

**Affiliations:** ^1^Novartis Pharmaceuticals Corporation, East Hanover, NJ, United States; ^2^Novartis Institutes for Biomedical Research, Basel, Switzerland; ^3^Novartis Institutes for Biomedical Research, Cambridge, MA, United States; ^4^Department of Biostatistics, Bioinformatics and Biomathematics, Georgetown University, Washington, DC, United States; ^5^Cambridge Cognition, Cambridge, United Kingdom; ^6^Neurotrack Technologies, Inc., Redwood City, CA, United States; ^7^Department of Medicine, School of Medicine, Stanford University, Stanford, CA, United States; ^8^ElMindA Ltd., Herzliya, Israel; ^9^Sonde Health, Inc., Boston, MA, United States; ^10^Groningen Institute for Evolutionary Life Sciences, University of Groningen, Groningen, Netherlands; ^11^Centre for Human Drug Research, Leiden, Netherlands; ^12^Department of Psychiatry, Leiden University Medical Center, Leiden, Netherlands

**Keywords:** digital biomarkers, major depression, mobile health, novel endpoints, variable selection

## Abstract

**Background:** Digital technologies have the potential to provide objective and precise tools to detect depression-related symptoms. Deployment of digital technologies in clinical research can enable collection of large volumes of clinically relevant data that may not be captured using conventional psychometric questionnaires and patient-reported outcomes. Rigorous methodology studies to develop novel digital endpoints in depression are warranted.

**Objective:** We conducted an exploratory, cross-sectional study to evaluate several digital technologies in subjects with major depressive disorder (MDD) and persistent depressive disorder (PDD), and healthy controls. The study aimed at assessing utility and accuracy of the digital technologies as potential diagnostic tools for unipolar depression, as well as correlating digital biomarkers to clinically validated psychometric questionnaires in depression.

**Methods:** A cross-sectional, non-interventional study of 20 participants with unipolar depression (MDD and PDD/dysthymia) and 20 healthy controls was conducted at the Centre for Human Drug Research (CHDR), the Netherlands. Eligible participants attended three in-clinic visits (days 1, 7, and 14), at which they underwent a series of assessments, including conventional clinical psychometric questionnaires and digital technologies. Between the visits, there was at-home collection of data through mobile applications. In all, seven digital technologies were evaluated in this study. Three technologies were administered via mobile applications: an interactive tool for the self-assessment of mood, and a cognitive test; a passive behavioral monitor to assess social interactions and global mobility; and a platform to perform voice recordings and obtain vocal biomarkers. Four technologies were evaluated in the clinic: a neuropsychological test battery; an eye motor tracking system; a standard high-density electroencephalogram (EEG)-based technology to analyze the brain network activity during cognitive testing; and a task quantifying bias in emotion perception.

**Results:** Our data analysis was organized by technology – to better understand individual features of various technologies. In many cases, we obtained simple, parsimonious models that have reasonably high diagnostic accuracy and potential to predict standard clinical outcome in depression.

**Conclusion:** This study generated many useful insights for future methodology studies of digital technologies and proof-of-concept clinical trials in depression and possibly other indications.

## Introduction

Depression is a common psychiatric disorder, with more than 264 million people affected worldwide (1). Symptoms of depression may manifest on multiple levels, including subjective emotional, cognitive, behavioral, and physical. There is currently a strong need for more efficient and valid monitoring of symptoms and drug treatment effects in depression. One problem in research and development of (novel) antidepressant treatments is the lack of objective, clinically relevant outcome measures. For instance, in major depressive disorder, conventional efficacy measures include the Hamilton Depression Rating Scale (HAM-D) (2) and the Montgomery-Åsberg Depression Rating Scale (MADRS) (3), which are subjective clinician rating scales. While these measures are well-established and broadly implemented, they tend to be administered infrequently (as single time point assessments), and are subject to rater bias and exhibit high variability, which translates into the need for large clinical trials to detect clinically meaningful treatment differences.

Digital technologies have the potential to provide more objective and precise tools to detect depression-related symptoms (4). Deployment of digital technologies in clinical research can enable remote collection of large volumes of clinically relevant data, which may be less burdensome than traditional in-clinic visits and may be more reflective of clinically relevant changes. In fact, high frequency data may be useful for detecting behaviors, objective prodromal signs or symptoms that would not have been captured using conventional rating scales or even noticed by the patients themselves. For instance, mobile applications to assess patient-reported outcomes (PROs) and performance outcomes (PerfOs) are becoming increasingly acceptable in clinical research to track changes in mood and cognition (5–7). Wearable technologies, such as smartwatches and novel sensors can generate useful digital biomarkers of depression in real-world settings (8–10). Furthermore, technologies based on high-density electroencephalogram (EEG) data can provide electrophysiological markers of depression that may be useful for both diagnostic and health monitoring purposes (11).

The present study was an exploratory, cross-sectional, naturalistic study to assess the utility of seven digital technologies in subjects with unipolar depression (MDD and PDD/dysthymia) and healthy controls. These technologies can be broadly categorized as mobile apps that provided data outside of the clinic (an interactive tool for the self-assessment of mood, and a cognitive test; a passive behavioral monitor to assess social interactions and global mobility; and a platform to perform voice recordings and obtain vocal biomarkers), and technologies that were evaluated in-clinic (a neuropsychological test battery; an eye motor tracking system; a standard high-density EEG-based technology to analyze the brain network activity during cognitive testing; and a task quantifying bias in emotion perception).

In the current study, the following research questions were of interest:

- Which technologies are useful to distinguish between depressed and healthy subjects?
- Can we build accurate classifiers (depressed vs. healthy) using parsimonious models with select digital biomarkers?
- Can we explain between-subject variation in MADRS (and possibly predict individual MADRS scores) using digital biomarker data?

This study provides preliminary estimates of classification accuracy of the digital technologies and describes digital biomarkers that could be useful for characterizing unipolar depression.

## Methods

### Study Design

This was a cross-sectional, non-interventional study, conducted at the Centre for Human Drug Research (CHDR) in the Netherlands. Forty participants (20 subjects with unipolar depression and 20 healthy controls; Table 1) were enrolled. Key inclusion criteria for all participants were: (1) male or female, 18–65 years inclusive; (2) must read and speak Dutch as first language and English as second language; (3) able to comply with the study procedures, prohibitions and restrictions (drug and alcohol use); and (4) Android-based smartphone user. Subjects with depression met the diagnostic criteria for at least one of the following disorders as confirmed with the Mini International Neuropsychiatric Interview (M.I.N.I.) (12): current major depressive disorder (MDD) without psychotic features according to DSM-5, or current persistent depressive disorder (PDD) or dysthymia according to DSM-5, which was corroborated by the attending general practitioner, psychiatrist or psychotherapist. Depression severity was moderate as reflected by a HAM-D total score of > 16 at screening. Depressed subjects with significant suicidality as demonstrated by the Columbia Suicide Severity Rating Scale (C-SSRS) (https://cssrs.columbia.edu), were only included if safety was not expected to be jeopardized during the study. The use of mono-aminergic antidepressant drugs at a stable dose for at least 4 weeks was allowed (6 weeks for fluoxetine). The full list of inclusion and exclusion criteria for both healthy subjects and patients can be found in the Supplementary Material.

**Table 1:** Demographic and baseline characteristic summary.

**Characteristic**	**Patients (*N* = 20)**	**Healthy controls (*N* = 20)**	**Total (*N* = 40)**
**Age (years)**			
Mean (SD)	35.0 (14.9)	27.5 (8.86)	31.2 (12.7)
Median	28.5	24.5	25.5
Range	20.0–63.0	20.0–52.0	20.0–63.0
**Sex -** ***n*** **(%)**			
Male	3 (15.0)	4 (20.0)	7 (17.5)
Female	17 (85.0)	16 (80.0)	33 (82.5)
**Race –** ***n*** **(%)**			
Asian	0 (0.0)	1 (5.0)	1 (2.5)
Black Or African American	0 (0.0)	1 (5.0)	1 (2.5)
Mixed	2 (10.0)	1 (5.0)	3 (7.5)
White	18 (90.0)	17 (85.0)	35 (87.5)
**Body Mass Index (kg/m**^**2**^**)**			
Mean (SD)	27.4 (6.73)	22.2 (2.45)	24.8 (5.64)
Median	26.2	22.1	24.2
Range	16.9–47.7	18.6–27.2	16.9–47.7
**MADRS total score**^**a**^			
Mean (SD)	25.6 (7.09)	1.2 (1.86)	13.4 (13.4)
Median	28.5	0.5	9.2
Range	10.0–36.7	0.0–8.3	0.0–36.7
**HAM-D total score**^**b**^			
Mean (SD)	20.4 (2.54)	1.2 (1.14)	10.8 (9.94)
Median	20.0	1.0	10.0
Range	17.0–25.0	0.0–3.0	0.0–25.0
**C-SSRS total score**^**c**^			
Mean (SD)	32.1 (20.8)	1.1 (2.69)	16.6 (21.4)
Median	26.5	0.0	7.0
Range	0.0–77.0	0.0–8.0	0.0–77.0

a *MADRS = Montgomery-Åsberg Depression Rating Scale. It was assessed at 3 study visits. For each participant, the MADRS total score was derived at each visit, and then the average value of the MADRS total score was obtained per subject*.

b *HAM-D = Hamilton Depression Rating Scale (at screening)*.

c *C-SSRS = Columbia-Suicide Severity Rating Scale (at screening)*.

**Figure 1:**
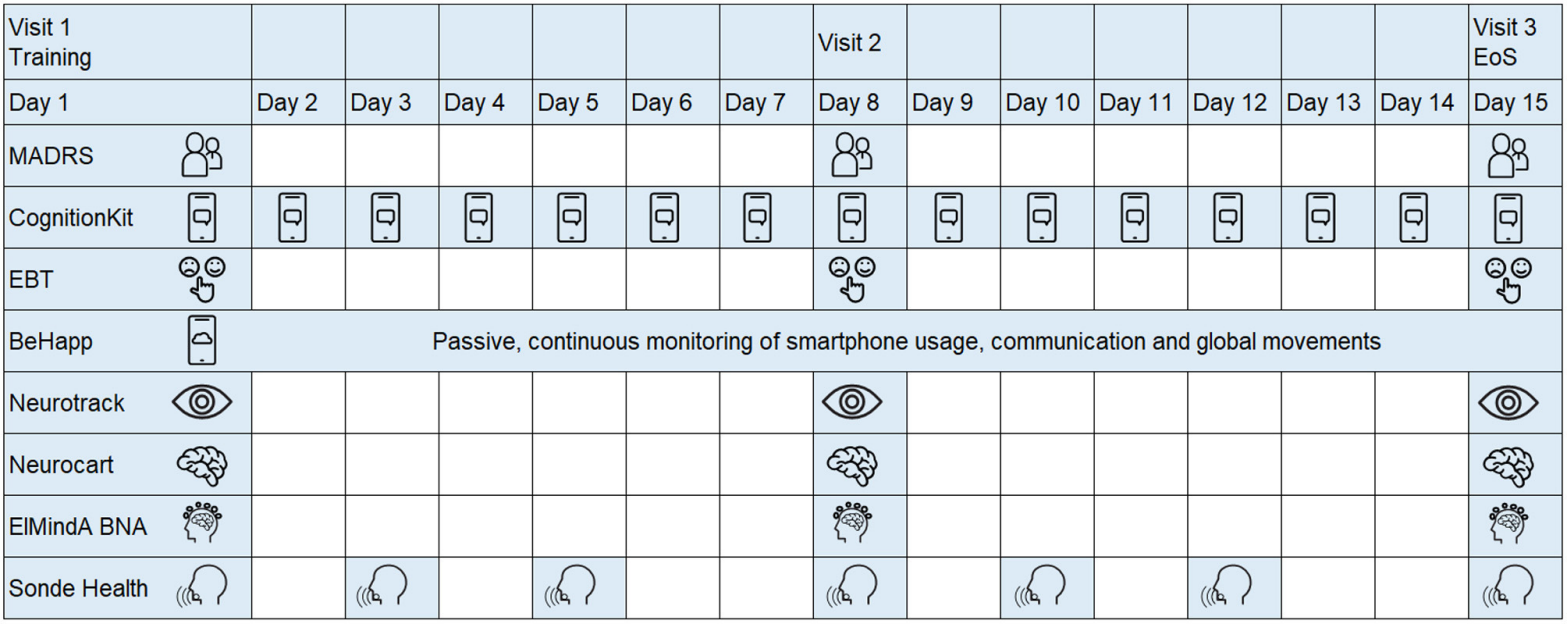
Study design schematic.

The study schematic is shown in Figure 1. Subjects who expressed interest in participating were asked to provide study-specific written informed consent and to attend a screening visit (from 21 to 2 days prior visit 1) to determine their eligibility. Eligible subjects were invited for three in-clinic visits at days 1, 7, and 14. At each visit, study participants underwent a series of assessments, including conventional psychometric questionnaires and digital technologies. Between the visits, there was at home collection of data through mobile applications. In all, seven digital technologies were evaluated (Table 2). Four technologies (Neurotrack, Neurocart, ElMindA BNA™, and Emotional Bias Task) were tested in-clinic only, whereas for the other three technologies (Cognition Kit, BeHapp, and Sonde Health), data were also collected outside the clinic. At visit 1, there were training sessions for all digital technologies and installation of mobile applications on the participants' smartphones. The details of the assessment schedule can be found in the Supplementary Material.

**Table 2:** Digital technologies assessed in the study.

**Technology**	**Description**	**Comment**	**References**
Cognition Kit	Mobile app that collects data from high frequency assessments of mood and cognition	Self-reported mood assessment (PHQ2) and cognitive test measure (dPrime)	(13, 14)
Emotional Bias Task (EBT)	The task evaluating whether there is a bias in emotion perception	Extent of emotional bias is quantified using *bias point*	(15)
BeHapp	Mobile behavioral tracker app to assess social interactions and global mobility	Features derived based on passive data collection and monitoring	(16–18)
NeuroTrack	Eye motor tracking system through web cameras within devices to assess cognitive processes	Neurophysiological assessments and a self-reported outcome (SMI score)	(19)
NeuroCart	A battery of minimally invasive neurophysiological and neuropsychological assessments	Saccadic eye movements, smooth pursuit eye movement, body sway pupil size ratio, Bond and Lader and Bowdle Visual Analog Scales (VAS)	(20)
ElMindA BNA™	EEG-based technology to analyze the brain network activity	Neurophysiological and neurocognitive assessments	(11)
Sonde Health	Voice-based technology to derive biomarkers of mental and physical health	Features derived from short speech samples based on voice characteristics	(21, 22)

Due to the exploratory nature of the study, the sample size was chosen to balance the considerations of feasibility and statistical efficiency. Utility of a given digital technology can be quantified by its ability to detect statistically significant differences between two groups. A sample size *n* = 40 (20 subjects with unipolar depression and 20 healthy controls) provides ~80% power of the two-sample *t*-test to detect statistically significant differences between the groups with a 10% two-sided significance level, if the true effect size (standardized difference of means for a continuous outcome measure) is 0.8. For smaller effect sizes, the power is lower; for instance, for the true effect size of 0.6 (or 0.5), the corresponding value of power is 0.59 (or 0.46). These calculations were done using nQuery sample size software (23).

### Data Collection and Feature Extraction

#### Cognition Kit

The Cognition Kit app analyzes and summarizes data from high-frequency assessments of mood and cognition. The technology has been validated in previous studies in both healthy subjects and patients with major depressive disorder (13, 14). In the current study, the participants received a daily reminder to complete the assessment of subjective mood and cognitive function. The cognitive test assessed working memory using the 2-Back, with the outcome measure dPrime (14), which is the ratio of hits (correct detection of a 2-Back match) to false alarms (response during no match). For mood assessment, two questions adapted from the Patient Health Questionnaire-9 (PHQ-9) (24) were administered to participants, with responses coded on a 4-point scale indicating the severity of the symptoms over the course of the day using a chat bot interface. A response to each question was scored from 0 to 3, with 3 representing the greatest severity of the symptom; i.e., Question 1: Little interest or pleasure in doing things? (rate: 0–3), and Question 2: Feeling down, depressed or hopeless? (rate: 0–3). A total score, PHQ2, was obtained as the sum of two responses on a scale of 0–6. Longitudinal data per subject (dPrime and PHQ2) were collected on a daily basis, each time the subject engaged with the app.

#### Emotional Bias Task

The EBT indicates whether there is a bias in emotion perception (15). Negative bias (the tendency to perceive ambiguous facial emotion expressions negatively, e.g., “sad”) is common in a range of mood disorders. During clinic visits participants viewed images of human faces that were morphed between happy and sad emotions of varied intensities, and they were instructed to indicate which emotion they perceive the face to be. There were 15 gradations of intensity for each emotion. The key outcome measure was the *bias point*, which represented the number of trials on which “happy” had been chosen as the label for the ambiguous facial expression the participants were presented with. A bias point of 15 indicated always selecting “happy” whereas a bias point of 7.5 indicates zero bias. The EBT was identified as a potentially promising technology for assessment of depression half-way through the study; therefore it was added in the protocol amendment and was evaluated only for the last 20 subjects in the study (*n* = 10 healthy and *n* = 10 patients with depression).

#### BeHapp

BeHapp is a digital phenotyping service aimed at passive monitoring of human subjects in formal (medical) scientific research. BeHapp aims to provide a “quantified” perspective on human behavior in terms of mobility and social interaction. The service has been applied to research the concept of social functioning in various studies on mental health amongst populations including participants suffering from schizophrenia, Alzheimer's disease and major depression (16–18). After activation, the BeHapp application remained active in the background of the participant's smartphone. Throughout the study duration, it tapped into various sources of behavioral data including communication events, phone usage logs (e.g., WhatsApp, Facebook), geographic location data, and Wi-Fi sensor data. Importantly, BeHapp is a research instrument that adheres to strict requirements toward privacy and informed consent that are common to formal scientific research projects involving human subjects (25). The data is end-to-end encrypted and the service consistently applies the principle of least privileges and zero trust. A total of 10 behavioral features were derived per subject. An example of a feature based on communication events is the mean usage time of communication apps. An example of a feature based on geographic location data is the total amount of time spent at home. A full list of BeHapp features can be found in the Supplementary Material.

#### Neurotrack

Neurotrack provides a system to track eye movements through web cameras within devices (laptops, tablets, and smartphones), which can be used to assess cognitive processes (19). Data from Neurotrack was ascertained at each in-clinic visit. The tasks included: (1) *Visual Paired Comparison (VPC)* – a participant is shown a series of paired images during a familiarization phase, and then is exposed to novel images. Two main outcome measures from the VPC are the *novelty preference*, calculated as percentage of time a participant is viewing the novel image, and *oscillation count*, calculated as the number of times participant switches from one image to the other. (2) *Self-reported Questionnaire* – a participant reports their current health related data. For each participant at a given visit, the derived parameters included mean and standard deviation of the novelty preference score calculated over 20 novelty performance trials; mean of the interstimuli oscillations over 20 trials; and the subjective memory impairment (SMI) score.

#### Neurocart

Neurocart® is a minimally-invasive, validated CNS test battery that quantifies functional CNS domains relevant to early drug development, and has been demonstrated to be sensitive to the effects of a vast array of CNS-penetrating compounds (20). Since it was developed primarily to confirm pharmacological activity of novel CNS drugs in early phase drug trials in healthy volunteers, it still remains to be established whether the functional CNS-related biomarkers that reflect pharmacological activity are relevant for patient populations with neuropsychiatric disease. Neurocart assessments were conducted at three in-clinic visits. At visit 1, there was only a training for the technology, whereas at each of the visits 2 and 3, the technology tests were administered three times—at 60, 160, and 230 min after admission. These tests could be classified into six categories; see also (20): (1) *Adaptive tracking test* measuring visuomotor coordination and sustained attention; (2) *N-back test* measuring working memory; (3) *Saccadic eye movements* to assess alertness and vigilance; (4) *Smooth pursuit eye movements* and *Body sway* to assess motor coordination; (5) *Pupillometry* as a measure for autonomic nervous system function; and (6) *Bond and Lader* and *Bowdle Visual Analog Scales (VAS)* as subjective measures. A total of 43 parameters per subject per visit were derived (average values were taken across the 3 time point assessments at a given visit).

#### ElMindA Brain Network Activation™

BNA™ is a novel, non-invasive, imaging technology, software only device that utilizes advanced algorithms to analyze the brain network activity from the recorded EEG data (11). EEG data were acquired at three in-clinic visits. At each visit, the EEG net recording time was ~1 h. Standard high-density EEG was performed while the subject was seated comfortably in a quiet room, in front of a computer monitor. The derived features could be grouped into three categories: (1) *Resting state EEG* (2–5 min with eyes closed while recording) – nine parameters in each of the alpha, beta, and gamma spectrum; (2) *Auditory Oddball (AOB) task* – 13 parameters providing different measures of sensory processing, working memory, and attention allocation; and (3) *Visual Go No-Go (VGNG) task* – 7 parameters, including filtering of information latency and amplitude, inhibitory control latency and amplitude, motor inhibition latency and amplitude, and accuracy (% of correct responses). In all, 47 features per subject per visit were derived.

#### Sonde Health Voice Analytics Technology

Voice analytics has shown promise for detecting symptoms of depression (21, 22). Study participants entered sound data through the smartphone application twice per week. The voice samples were collected from different tasks, such as sentence/passage reading, free speech response to a specific prompt, and the Stroop task (e.g., the participant is instructed to read out loud the color of the word presented on the screen). Each voice sample, typically a.wav file, was represented by a non-static time series of thousands of vocal features. Signal processing algorithms were applied on a per-file basis to extract various features (e.g., pitch) from various time windows within a sample of speech (e.g., average pitch within the first 20 milliseconds of speech). For the purpose of data analysis, a total of 72 features were derived per subject.

### Data Analysis

The analysis was performed separately for each technology, and included the following steps: exploratory analysis, classification analysis, and regression modeling. Since the study period was only 2 weeks, the participants within each group (healthy or depressed) were expected to be in stable condition. Therefore, as a general rule we took averages of relevant valid longitudinal measurements within subject to derive individual features per subject. Any observation marked as low data quality was excluded from the analysis. No missing data imputation was done.

#### Exploratory Analysis

For each technology, digital biomarker features were explored using summary statistics and graphically, by group (healthy or depressed). Pairwise correlations among relevant features and total MADRS score were estimated.

#### Classification

For a given technology, a classification problem can be formulated as follows. Let *Y*∈ {0, 1} denote the group indicator for the subject (*Y* = 1, if depressed; *Y* = 0, if healthy). Let **X** = (*X*_1_, …, *X*_*m*_) denote a vector of digital biomarkers for the technology (*X*_*j*_ may represent some derived summary measure, such as mean, SD, percentile, etc.). For a suitably chosen function *g*(**X**) and a cutoff value *c*, a subject with digital readouts **x** = (*x*_1_, …, *x*_*m*_) is classified as depressed, if *g*(**x**) ≥ *c*; or as healthy, of *g*(**x**) < *c*. Various classification methods are available (26). In our analysis, we implemented two approaches: (1) logistic regression; and (2) an approach based on the predicted MADRS score using a multiple linear regression model with selected digital biomarkers.

A logistic model has the form $\log\left(\frac{p\left(\text{x},\beta \right)}{1-p\left(\text{x},\beta \right)}\right)=\beta_{0}+\overset{m}{\underset{i=1}{\sum }}\beta_{i}x_{i}$, where **β** = (β_0_, β_1_, …, β_*m*_) are model parameters and *p* (**x**, **β**) = Pr (*Y* = 1|**x**) is the probability that a subject with a vector of digital biomarkers **x** = (*x*_1_, …, *x*_*m*_) belongs to the depression group. Given an estimate $\hat{\beta }$ of **β**, a subject with digital readouts $\tilde{\text{x}}$ would be classified as depressed, if $p\left(\tilde{\text{x}},\hat{\beta }\right)>0.5$; or as healthy otherwise. In the described approach, it is assumed that the logistic model is based on the selected set of digital biomarkers **X** = (*X*_1_, …, *X*_*m*_).

We searched for parsimonious models that can be easily interpreted and contain only those biomarkers that truly contribute to the accuracy of a classifier. For this purpose, we applied a stepwise variable selection method for model building, with a threshold for significant predictors as *p* < 0.1. The analysis included all participants with valid data for a given technology. Statistical properties (accuracy) of the resulting classifier were obtained using leave-one-out cross validation (LOOCV), which is known to reduce the misclassification rates when the classifiers are estimated and used on the same dataset (26).

The performance of a classifier was assessed by calculating sensitivity, specificity, and overall classification accuracy. We also constructed the receiver operating characteristic (ROC) curves [plots of sensitivity vs. (1–specificity) for different threshold classification values] and calculated the area under the curve (AUC) values.

#### Linear Regression Modeling

For each technology, we estimated the relationship between total MADRS score and digital biomarkers using multiple linear regression. Parsimonious models with most significant predictors obtained through stepwise variable selection procedures were sought. Quality of fitted models was assessed graphically, using plots of observed vs. predicted MADRS total scores and using model residual plots. The proportion of variance in MADRS total scores explained by the model (adjusted *R*^2^) was derived.

In addition, classifiers based on model-predicted MADRS were obtained as follows. Suppose a linear regression model is fit as $E(MADRS)=\gamma^{\prime }\text{x}=\gamma_{0}+\sum_{i=1}^{m}\gamma_{i}x_{i}$, where **γ** = (γ_0_, γ_1_, …, γ_*m*_) are model parameters and **x = **(1, *x*_1_, …, *x*_*m*_) is a set of digital biomarkers, including intercept. Given an estimate $\hat{\gamma }$ of **γ** and a vector of digital readouts $\tilde{\text{x}}=\left(1,{\tilde{x}}_{1},\ldots ,{\tilde{x}}_{m}\right)$, the estimated MADRS total score, $\hat{MADRS}={\hat{\gamma }}_{0}+\overset{p}{\underset{i=1}{\sum }}{\hat{\gamma }}_{i}{\tilde{x}}_{i}$ is compared against an established clinical cutoff of 10.5 points (27). A subject is classified as depressed, if $\hat{MADRS}\geq 10.5$; or as healthy, if $\hat{MADRS}<10.5$. The diagnostic accuracy of this classifier was assessed by calculating sensitivity, specificity, and overall classification accuracy using LOOCV. The ROC curves for different values of a classification threshold were constructed, and the corresponding AUC values were calculated.

## Results

### Subject Demographics and Psychometric Scores

**Figure 2:**
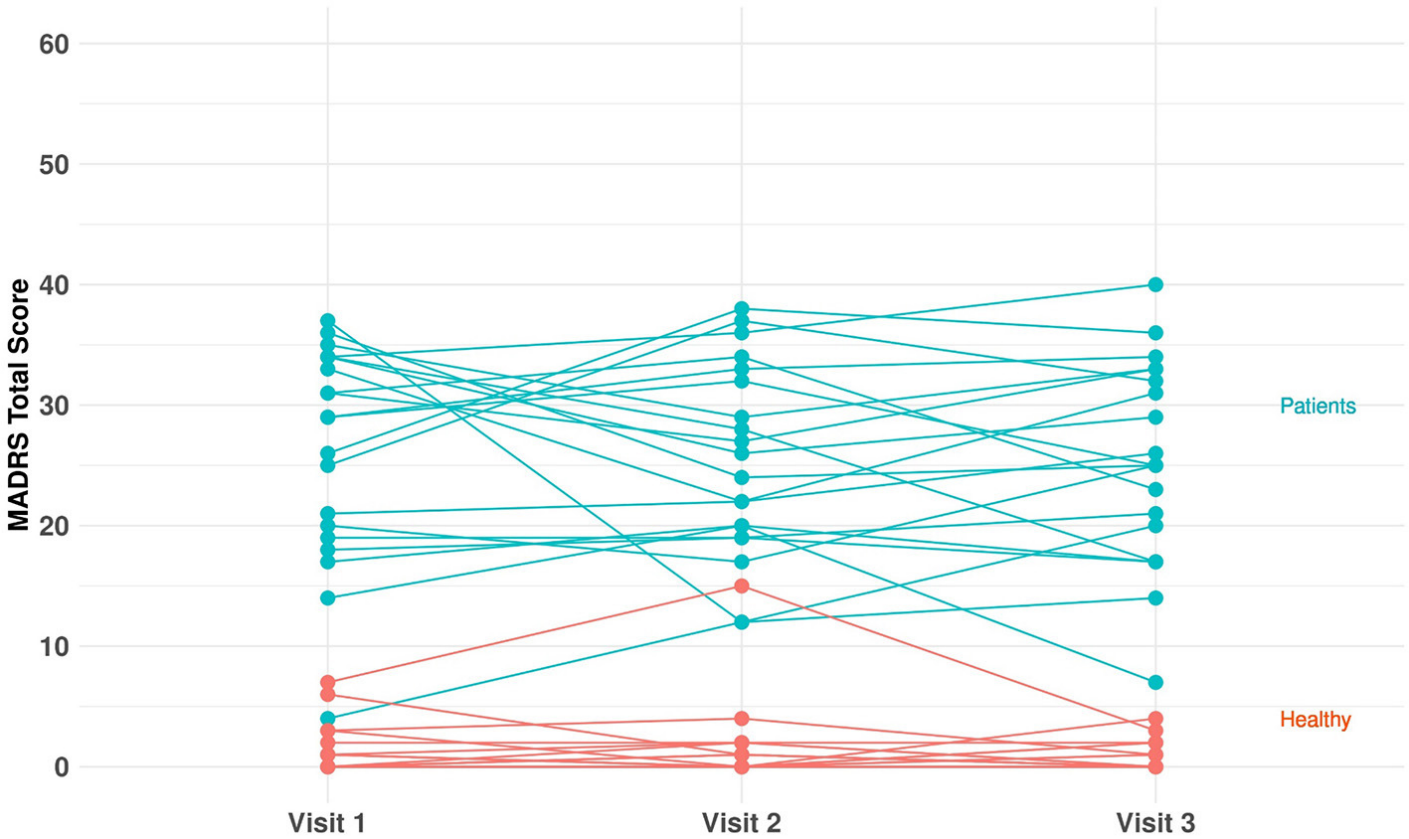
MADRS total score per group (healthy and unipolar depression) and per visit.

In total, 40 subjects (20 MDD and PDD/dysthymia and 20 healthy) were enrolled and completed the study. Table 1 provides a summary of key demographic and baseline characteristics. The majority of subjects were female (82.5%) and white (87.5%). The mean age was 31.2 (range: 20–63) years, and the mean BMI was 24.8 (range: 16.9–47.7) kg/m^2^. The mean (range) of HAM-D total score at screening was 20.4 (17–25) for the patients, and it was 1.2 (0–3) for the healthy controls. The mean (range) of C-SSRS total score at screening was 32.1 (0–77) for the patients, and it was 1.1 (0–8) for the healthy controls. Figure 2 shows individual MADRS total scores per group and per visit. For each participant, we derived the average value of the MADRS total score across the visits and obtained the corresponding summary statistics per group. The group mean (range) of the MADRS total score was 25.6 (10.0–36.7) for the patients and 1.2 (0–8.3) for the healthy controls (Table 1).

Participation in this study was without health-related intervention. A treating physician was solely responsible for determining any therapeutic strategy for patients with depression. Supplementary Figure 1.1 shows the individual values of duration of the antidepressant medication (range: 1.4 months−26.7 years; median = 18.7 months−1.6 years). All but two patients had Serotonin Reuptake Inhibitor (SRI)-based antidepressant therapy. Two patients had Tricyclic Antidepressants (TCAs). Furthermore, there was one patient who had an SRI-based primary therapy and a Norepinephrine and Dopamine Reuptake Inhibitor (NDRI)-based secondary therapy.

### Exploratory Analysis

Details of the exploratory analyses can be found in the Supplementary Material. From the plots of pairwise correlations, one important finding is that some subjective (self-reported) outcomes had strong positive correlation with MADRS total score. For instance, the PHQ2 component of Cognition Kit and the subjective memory impairment (SMI) score of Neurotrack each had a correlation of *r*= 0.9 with MADRS total score.

Furthermore, MADRS total score had moderate negative correlation with some features of the behavioral tracker app (BeHapp). Subjects with higher MADRS total score tended to have lower average distance from home (*r* = −0.25), lower entropy of the usage time of communication apps (*r* = −0.31), lower total count of communication apps usage (*r* = −0.42), and lower number of WhatsApp usage (*r* = −0.43). One may conjecture that higher depression severity is associated with lower social activity.

For the resting state EEG data, we observed high positive correlations (*r* = 0.8–1.0) among pairs of variables in the alpha power and gamma power spectra. Also, MADRS total score had a moderate negative correlation (*r* = −0.3 to −0.4) with variables in the alpha power spectrum and it had relatively low correlations with the variables from the auditory oddball (AOB) task and visual go-no-go (VGNG) task.

The exploratory analysis also revealed that not all self-reported outcomes provided evidence of a between-group difference. For instance, for PHQ2 and dPrime (two key features of the Cognition Kit) there was a clear separation between healthy and depressed groups with respect to PHQ2 but not dPrime. In addition, there was evidence of a learning effect – an increasing trend in dPrime over time, which is consistent with some previous research findings in major depression (13).

### Classification Analysis

Table 3 shows diagnostic accuracy of classifiers based on the logistic model and the linear model-predicted MADRS total score using different digital technology features. For the logistic classifier, the rule was as follows: classify a subject as depressed, if the model-estimated probability that this subject has unipolar depression is >50%; or as healthy otherwise. For the linear model classifier, a subject was classified as depressed, if their model-predicted MADRS total score was ≥10.5; or as healthy otherwise.

**Table 3:** Classification analysis using logistic model and linear model-predicted MADRS using different digital technology features.

	**Logistic model**			**Linear model-predicted MADRS**		
**Digital technology features**	**Sensitivity^**a**^**	**Specificity^**b**^**	**Accuracy^**c**^**	**Sensitivity^**a**^**	**Specificity^**b**^**	**Accuracy^**c**^**
PHQ2 (Cognition Kit)	1.0 (19/19)	0.95 (18/19)	0.97 (37/38)	0.95 (18/19)	1.0 (19/19)	0.97 (37/38)
Subjective memory impairment (SMI) (Neurotrack)	0.90 (18/20)	1.0 (19/19)	0.95 (37/39)	0.90 (18/20)	1.0 (19/19)	0.95 (37/39)
Neurophysiological features (Neurocart)	0.90 (18/20)	0.80 (16/20)	0.85 (34/40)	0.75 (15/20)	0.85 (17/20)	0.80 (32/40)
Behavioral tracker (BeHapp)	0.64(9/14)	0.64 (9/14)	0.64 (18/28)	0.86 (12/14)	0.64 (9/14)	0.75 (21/28)
EEG – resting state (ElMindA)	0.70 (14/20)	0.70 (14/20)	0.70 (28/40)	0.75 (15/20)	0.70 (14/20)	0.73 (29/40)
EEG – cognitive tasks (ElMindA)	0.70 (14/20)	0.50 (9/18)	0.61 (23/38)	0.80 (16/20)	0.44 (8/18)	0.63 (24/38)
Voice (Sonde)	0.60 (12/20)	0.70 (14/20)	0.65 (26/40)	0.70 (14/20)	0.55 (11/20)	0.63 (25/40)
Emotional bias task (EBT)	0.80 (8/10)	0.50 (5/10)	0.65 (13/20)	0.80 (8/10)	0.50 (5/10)	0.65 (13/20)

a *Sensitivity = proportion of patients with depression that are correctly identified as such (number who are patients with depression and are classified by the technology as depressed/number who are patients with depression)*.

b *Specificity = proportion of normal healthy subjects that are correctly identified as such (number who are normal healthy subjects and are classified by the technology as healthy/number who are normal healthy subjects)*.

c *Accuracy = overall proportion of correct classifications (number of correct classifications/total number of subjects)*.

For ElMindA BNA analysis, two separate models were built – one using resting state EEG features, and the other one using EEG cognitive task features (BNA) as predictors. This was done to better understand and interpret the added value of these two sets of features.

From Table 3, as expected, the overall highest classification accuracy (≥95%) was achieved using the LOOCV-classifiers based on the subject-reported outcome features (PHQ2 score of Cognition Kit and SMI score of Neurotrack), which were highly correlated with severity of depressive symptoms. The LOOCV-classifiers based on features from the neurophysiological test battery (Neurocart) were 80–85% accurate. For all other classifiers, the overall accuracy was in the range 61–75%. Note the different sample sizes (denominators) – for some technologies fewer than 40 subjects provided valid data for the analysis.

### Regression Analysis

**Figure 3:**
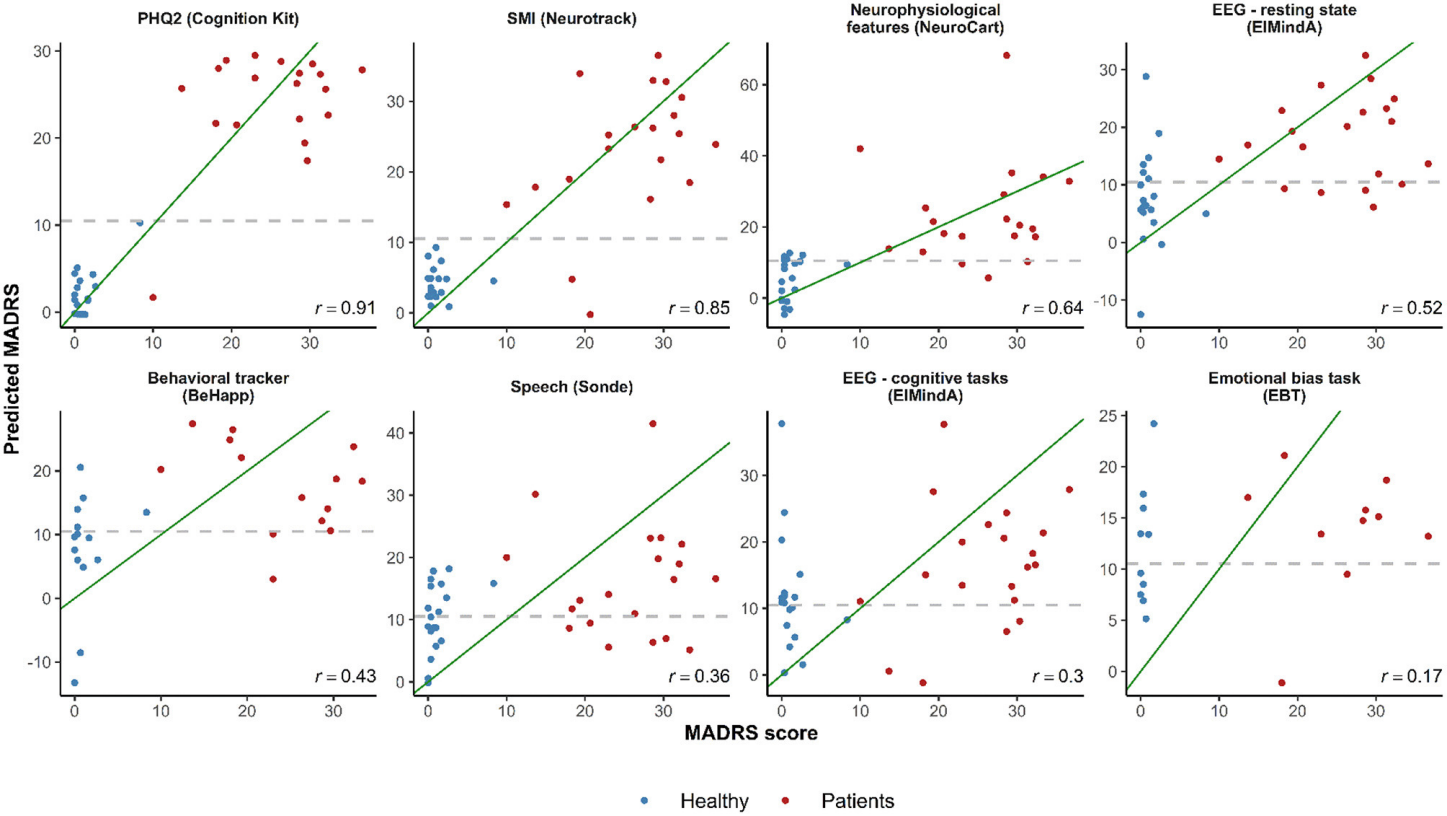
Observed vs. predicted average total MADRS score using different digital technology features. The green diagonal line represents a perfect match between observed and predicted MADRS total scores. In case of a strong linear relationship between MADRS total score and selected features, the observations are expected to fall along the green diagonal line. The gray dashed horizontal line at 10.5 represents a classification threshold: when predicted MADRS total scores for healthy subjects are above 10.5 or similar values for depressed subjects are below 10.5, these observations would be misclassified based on the linear model classifier. PHQ2 is a self-reported score of mood assessment. SMI is a subjective memory impairment score.

Figure 3 shows plots of observed vs. predicted MADRS total scores for the LOOCV-models using different technology features, and the corresponding Pearson's correlation coefficient calculated between predicted and real MADRS total score values. The three models with the highest correlation were based on subject-reported features: (1) PHQ2 (linear and quadratic terms) (*r* = 0.91); (2) SMI (*r* = 0.85); and (3) a model with three predictors (Bond and Lader lethargic–energetic VAS, Bond and Lader interested–bored VAS, and the outcome of the 2-Back working memory task) (*r* = 0.64). For other models, the correlation values were in the range from 0.17 to 0.52.

In the plots of Figure 3, in case of a linear relationship between MADRS total score and the selected features, the observations would be expected to fall close along the diagonal line. One can see cases when predicted MADRS total score values for healthy controls are above the 10.5 horizontal threshold line, and similar values for depression patients are below the 10.5 threshold. These observations would be misclassified based on the linear model classification rule we described earlier (cf. Table 3).

### Receiver Operating Characteristics Curves

**Figure 4:**
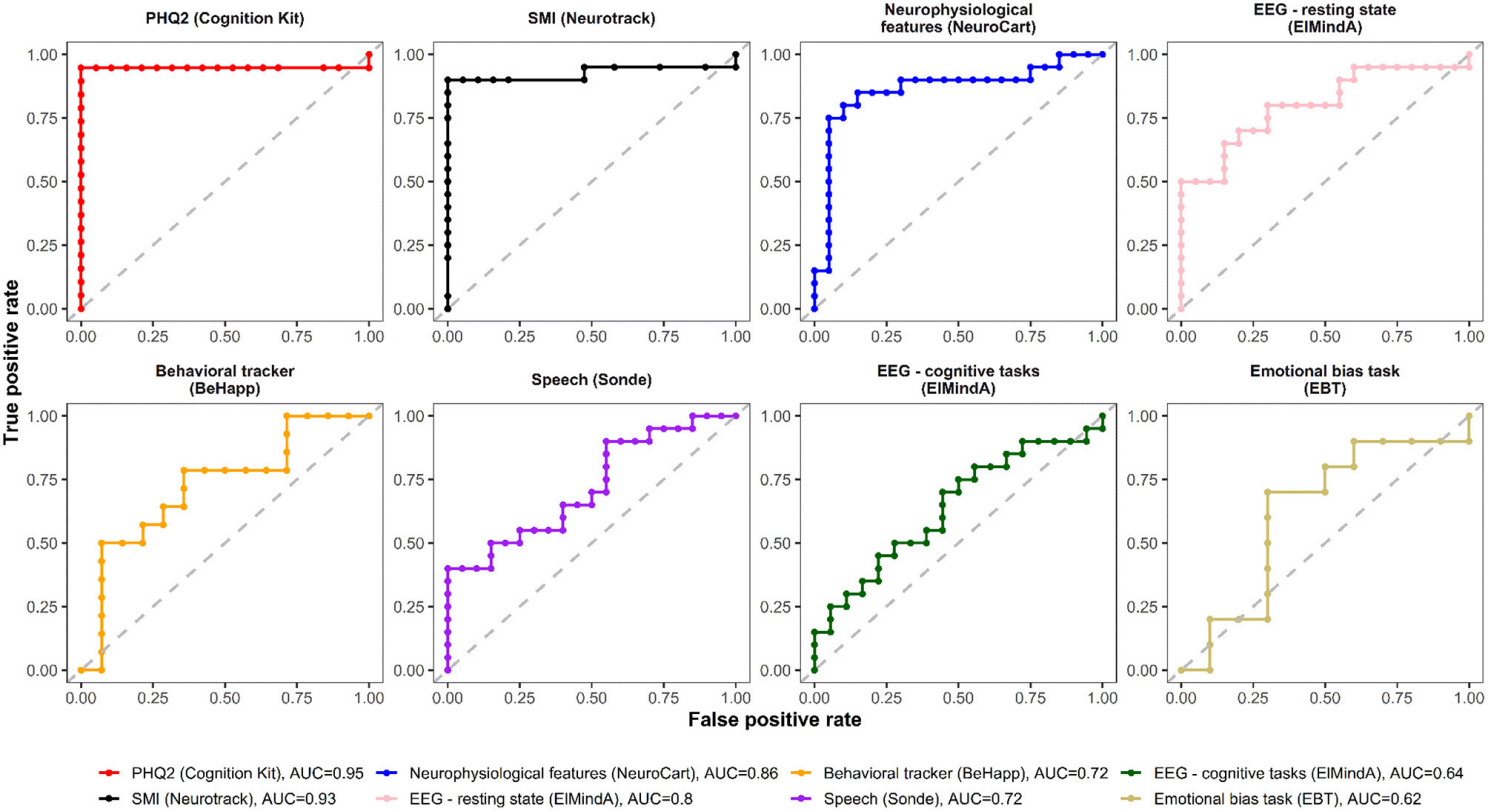
ROC curves of classifiers based on logistic regression using different digital technology features.

**Figure 5:**
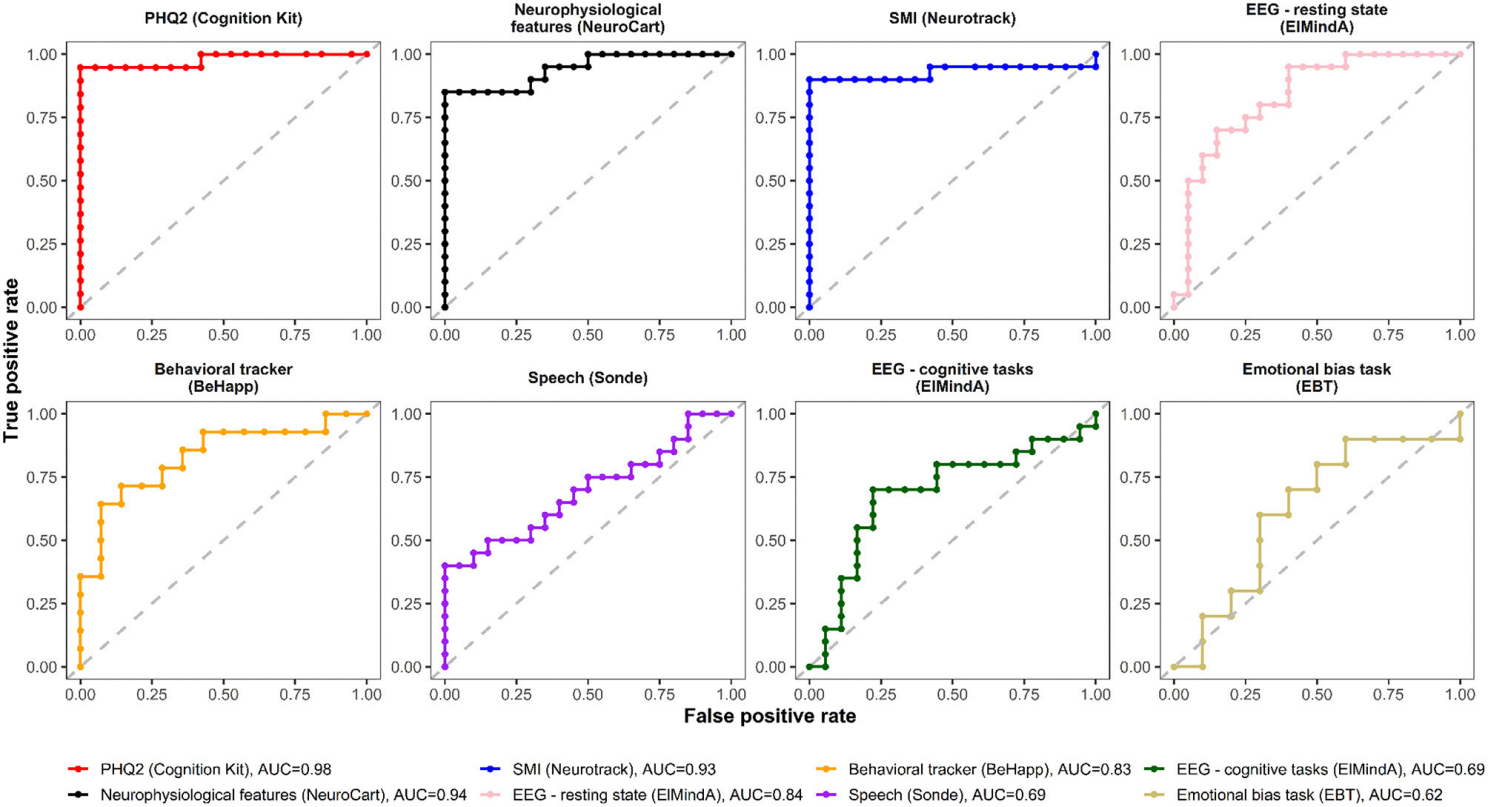
ROC curves of classifiers based on linear model-predicted MADRS using different digital technology features.

Figures 4, 5 are, respectively, the ROC curves for classifiers based on logistic regression and classifiers based on linear model-predicted MADRS total score using different technology features. For five models (PHQ2 of Cambridge Cognition; behavioral tracker features of BeHapp; neurophysiological features of Neurocart; EEG–resting state features of ElMindA; and EEG–BNA features of ElMindA), linear model-based classifiers (Figure 5) had somewhat higher values of ROC AUC compared to the corresponding logistic model-based classifiers (Figure 4). Two models had the same AUC values for both linear and logistic model-based classifiers: AUC = 0.93 for SMI of Neurotrack and AUC = 0.62 for EBT of Cambridge Cognition. For the speech features of Sonde Health, the logistic model-based classifier had AUC = 0.72 whereas the linear model-based classifier had AUC=0.69.

## Discussion

In this study, we investigated the utility of several novel digital technologies for characterizing depression in twenty participants with unipolar depression (MDD and PDD/dysthymia) and twenty healthy controls. Some technologies acquired data remotely, whereas for the other technologies data were collected during in-clinic visits.

One objective was to build parsimonious models to distinguish between depression patients and healthy controls using digital biomarker features. Some models were very good for this purpose – e.g., models based on subject-reported outcomes (PHQ2 of Cambridge Cognition and SMI score of Neurotrack) had ≥95% overall classification accuracy using LOOCV-logistic regression models. Not only did these features have high discriminatory power, but also they were found to be highly correlated with the MADRS total score, which is considered a conventional clinical endpoint in depression research. Through linear regression modeling, we were able to predict individual MADRS total scores using selected digital biomarker features, and use these models as classifiers. We found that linear model-based classifiers may improve diagnostic accuracy by several percentage points compared to logistic model-based classifiers; however, for some technologies (e.g., Neurocart and Sonde), the logistic model classifiers were slightly more accurate than the linear model classifiers. An additional merit of the developed linear models is that they may help to quantify and relate the magnitude of expected change in MADRS total score to the change in digital phenotypes. Larger studies will be required to further validate these findings.

Our data analysis was organized by technology – this was done to better understand individual features of different technologies and identify digital biomarker features that are most correlated with standard clinical assessments of depression. As the next step, we are planning to explore combinations of technologies for possible synergy and improvement of classification accuracy. A practical question is: data from which technologies should be combined? For those that are already very accurate any additional benefit (say, an increased classification accuracy from 95 to 98%) would be deemed as marginal. However, going from 80% accuracy to 90% accuracy or above could be quite an improvement. This is work in progress, beyond the scope of the current report. Another important note is that in this paper we presented mainly on the results of logistic regression and linear regression. Other supervised learning techniques, such as linear discriminant analysis and support vector machines, as well as unsupervised learning (different cluster analysis methods) may be useful and are under investigation.

In this study, we assessed correlations between various digital biomarker features and a clinical endpoint (MADRS total score). As MADRS is a subjective outcome measure, it is not surprising, that technologies with integrated subjective patient reported outcomes (e.g., PHQ2 of Cognition Kit; SMI score of Neurotrack) were found to correlate more strongly and predict MADRS total scores more accurately than other types of technologies. However, in contrast to MADRS, one of the main advantages of technologies such as Cognition Kit is the low-burden, remote, self-administration of subjective outcome measures. This advantage is underscored by the global pandemic of COVID-19, which has led to an unprecedented need for decentralized and virtual clinical trial settings. Another important advantage of this technology is repeatability. Daily administration of Cognition Kit demonstrated daily fluctuations in mood. Being able to measure daily fluctuations in clinical symptoms is important as this may allow more accurate monitoring of drug treatment effects and may additionally identify novel clinically relevant outcome measures, such as “number of days in low mood” or “weekly mood variability.” These types of outcome measures cannot be derived from conventional single time-point in-clinic assessments or with other more burdensome technologies.

This study provided important insights into digital phenotyping of depressed patients and healthy controls based on social interactions and global mobility data acquired through a behavioral tracker technology (BeHapp). Our findings suggest that depression may be associated with decreased communication via smartphone as well as less movement (more home stay). There was evidence of moderate correlations between some of BeHapp features and MADRS total score, and a linear model-based classifier had 75% overall classification accuracy (albeit it did not utilize the full sample size). We make two important notes on evaluation of the performance of behavioral trackers. First, our study was only 2 weeks long whereas quantifying human behavior takes time. Second, “social circumstances” of participants were not collected systematically, which could have provided powerful co-factors for analysis (e.g., if participants were employed or not). Hence, the BeHapp findings should be interpreted with caution. This type of technology holds promise of completely passive, low-burden outcome measures for clinical trials and calls for more extensive evaluation in larger and longer studies. From a technical perspective, BeHapp data flows are processed through systems that are carefully designed with respect to the privacy and security of the clinical research participants (25).

We also note that both the digitally acquired subjective outcome measures (such as PHQ2 of Cognition Kit or SMI score of Neurotrack) and the digital phenotyping features based on the behavioral tracker technology (BeHapp) should be distinguished from digital neurophysiological and neurocognitive markers, which may be less correlated with standard clinical questionnaires such as MADRS, and yet which may provide additional clinically relevant characteristics of depression. Due to the complexity of major depression, evaluating it with additional objective digital tools (such as EEG-based ElMindA BNA™ technology and voice-based Sonde Health technology) may help to quantify certain aspects that are not measured with traditional subjective clinical assessments.

Overall, the approach we took for design and analysis of this study may be applicable in other settings where both in-clinic and real-world digital data are collected, and there is a need to establish meaningful links between the two. By considering digital technologies (and their combinations), one can have low-burden, ecologically attractive digital phenotype assessments that could augment the conventional clinician interviews and provide additional clinically important information.

Our study had several limitations. The sample size was small and for each participant the study duration was only 2 weeks. No age or gender matching was done between healthy and depressed subjects, and there were more females than males in the study. We explored statistical models adjusting for age and gender; however in most instances these factors were found to be insignificant; this may be because of small sample sizes. The results of these additional analyses are not reported here, but are available upon reasonable request. Subjects with depression were on different antidepressant medications, and had varying durations of both antidepressant therapy and active illness. Although it therefore might be argued that these factors were potential confounders, we are of the opinion that such heterogeneity of the depressed sample actually reflects the clinical characteristics of non-clinical trial depressed populations quite well and therefore can be considered reflective of a real-world depressed population. In addition, it is important to point out that antidepressant treatment needed to be stable for at least 4 weeks prior to inclusion and remain unchanged for the duration of the study, which limited symptom fluctuation due to current treatment. Also, due to the non-interventional nature of this study, we were unable to address an important question of how sensitive the digital technologies are to measuring treatment effects. Finally, because the study was cross-sectional, it does not inform us about the usability of these technologies to monitor changes in depression symptom severity over time. Therefore, other types of studies with digital technologies are warranted before it can be inferred whether these technologies can be broadly applied in clinical trials as ultimately intended.

## Conclusion

In this study, we investigated seven digital technologies and identified promising digital biomarker features that correlate well with the depressive symptoms. We developed statistical models with selected digital features that have reasonably high diagnostic accuracy and potential to predict standard clinical outcome in depression. This study generated many insights that may be useful for future methodology studies of digital technologies and proof-of-concept clinical trials in depression and other indications.

## Data Availability Statement

The datasets presented in this article are not readily available because of the informed consent and confidentiality restrictions. Requests to access the datasets should be directed to alex.sverdlov@novartis.com.

## Ethics Statement

The study was reviewed and approved by Stichting Beoordeling Ethiek Biomedisch Onderzoek (BEBO), Assen, the Netherlands. All participants provided written informed consent prior to study participation.

## Author Contributions

OS, JC, KH, AD, BG-M, KB, MD, KR, and J-HC designed the study. OS, JC, LG, VDL, FA, BZ, JP, and AD analyzed the data. JC, KH, VD, VV, FC, JJA, NTB, ZP, GI, OL, DJ, RRJ, NJ, MJK, AZ, RZ, KR, ZZ, and GJ contributed to the data collection and the conduct of the study. OS wrote the first draft of the manuscript. All authors reviewed and approved the manuscript.

## Conflict of Interest

OS, JC, KH, LG, VD, FA, JP, VV, AD, BG-M, KB, MD, and J-HC were employed by Novartis. FC was employed by Cambridge Cognition. JA was employed by Neurotrack Technologies, Inc. ZP, GI, and OL were employed by ElMindA, Ltd. DJ was employed by Sonde Health, Inc. AZ, RZ, KR, ZZ, and GJ were employed by CHDR. The remaining author declares that the research was conducted in the absence of any commercial or financial relationships that could be construed as a potential conflict of interest.
