# Supplementary material for: A Study of Novel Exploratory Tools, Digital Technologies, and Central Nervous System Biomarkers to Characterize Unipolar Depression
Source: Front Psychiatry. 2021 May 6;12:640741. doi: 10.3389/fpsyt.2021.640741 (PMC8136319; doi:10.3389/fpsyt.2021.640741)
Supplement: Supplementary file 1 [file Data_Sheet_1.pdf]

Supplementary Material

This document contains additional information and analyses for the manuscript “A study of novel exploratory tools, digital technologies and CNS biomarkers to characterize unipolar depression”.

Contents

Supplementary Material 1
- Study Inclusion and Exclusion Criteria 3
  - Healthy Subject Inclusion Criteria 3
  - Healthy Subject Exclusion Criteria 3
  - Patient Inclusion Criteria 4
  - Patient Exclusion Criteria 4
  - General Restrictions 5
- Assessment Schedule 6
  - Table 1.1: Overall assessment schedule 6
  - Table 1.2: Detailed assessment schedule at Visit 2 and Visit 3 7
- Patient Characteristics 7
  - Figure 1.1: Duration of the antidepressant medication for the study patients 7
- Cognition Kit 8
  - Figure 2.1: Individual profiles and mean (SD) of PHQ2 vs. study day 8
  - Figure 2.2: Individual profiles and mean (SD) of dPrime vs. study day 9
  - Figure 2.3: Pairwise correlations of Cognition Kit features and MADRS 9
  - Table 2.1: Cambridge Cognition - Results of a logistic regression analysis 9
  - Table 2.2: Cambridge Cognition - Results of a linear regression analysis of MADRS 9
- Neurotrack 10
  - Figure 3.1: Mean (SD) of Neurotrack features vs. visit 10
  - Figure 3.2: Pairwise correlations of Neurotrack features and MADRS 11
  - Table 3.1: Neurotrack - Results of a logistic regression analysis 11
  - Table 2.2: Neurotrack - Results of a linear regression analysis of MADRS 11
- Neurocart 12
  - Figure 4.1: Pairwise correlations of Neurocart features and MADRS 13
  - Table 4.1: Neurocart - Results of a logistic regression analysis 14
  - Table 4.2: Neurocart - Results of a linear regression analysis of MADRS 14
- BeHapp 15
  - Figure 5.1: Pairwise correlations of BeHapp features and MADRS 16
  - Table 5.1: BeHapp - Results of a logistic regression analysis 16
  - Table 5.2: BeHapp - Results of a linear regression analysis of MADRS 16
- ElMindA 17

Figure 6.1: Pairwise correlations of ElMindA features and MADRS 19
Table 6.1: ElMindA - Results of a logistic regression analysis 20
Table 6.2: ElMindA - Results of a linear regression analysis of MADRS 21
Sonde Health 22
Figure 7.1: Pairwise correlations of Sonde features and MADRS 23
Table 7.1: Sonde - Results of a logistic regression analysis 23
Table 7.2: Sonde - Results of a linear regression analysis of MADRS 24
Emotional Bias Task (EBT) 24
Figure 8.1: Pairwise correlations of EBT features and MADRS 25
Table 8.1: EBT - Results of a logistic regression analysis 25
Table 8.2: EBT - Results of a linear regression analysis of MADRS 25

Study Inclusion and Exclusion Criteria

Subject selection was established by checking through all eligibility criteria at Screening and Visit 1. A relevant record (e.g. checklist) of the eligibility criteria was stored with the source documentation at the study site.

Healthy Subject Inclusion Criteria

Subjects eligible for inclusion in this study have to fulfill **all** of the following criteria:

1. Written informed consent must be obtained before any assessment is performed.
2. Male or female subjects, 18 to 65 years (inclusive).
3. Body mass index (BMI) between 18 and 30 kg/m2, inclusive at Screening.
4. Must read and speak Dutch as first language, and English as second language on secondary school diploma level.
5. Able to comply with the study procedures, prohibitions and restrictions (drug and alcohol use) as specified in the protocol.
6. Android-based smartphone.

Healthy Subject Exclusion Criteria

Subjects fulfilling **any** of the following criteria are not eligible for inclusion in this study:

1. Current or previous clinically relevant history or family history of psychiatric disorders, neurological disorders or neurosurgery.
2. Positive urine test for drugs of abuse at Screening or on study days or a current diagnosis of substance use disorder (including alcohol but excluding nicotine) or previous substance use disorder (including alcohol but excluding nicotine) within the past 12 months according to DSM-5.
3. Evidence of renal, hepatic, cardiovascular or metabolic dysfunction or any active or chronic disease or condition that could interfere with the conduct of the study, or that would pose an unacceptable risk to the subject in the opinion of the investigator (following a detailed medical history, physical examination, vital signs (systolic and diastolic blood pressure, pulse rate, body temperature) and12-lead electrocardiogram (ECG)). Minor deviations from the normal range ay be accepted, if judged by the investigator to have no clinical relevance.
4. Clinically significant abnormalities, as judged by the investigator, in laboratory test results (including hepatic and renal panels, complete blood count, chemistry panel and urinalysis). In the case of uncertain or questionable results, tests performed during Screening may be repeated before inclusion to confirm eligibility or to assist in evaluating clinical relevance.
5. Use of any medications (prescription or over-the-counter [OTC]), within 14 days of Visit 1, or less than 5 half-lives (whichever is longer). An exception is paracetamol (up to 2 g/day). Other exceptions will only be made if the rationale is clearly documented by the investigator.
6. Positive urine β-human chorionic gonadotropin (β-hCG) pregnancy test at Screening in women of childbearing potential.
7. Current enrollment in an interventional study.

Patient Inclusion Criteria

Patients eligible for inclusion in this study have to fulfill **all** of the following criteria:

1. Written informed consent must be obtained before any assessment is performed.
2. Males and females, age 18 to 65 years (inclusive).
3. Subjects must be diagnosed by the attending general practitioner, psychiatrist or clinical psychologist with, and meet the diagnostic criteria for at least one of the following disorders as confirmed with the Mini International Neuropsychiatric Interview (MINI):
    - Current major depressive disorder (MDD) without psychotic features according to DSM-5 (296.22, 296.23, 296.32, 296.33).
    - Current persistent depressive disorder (PDD) or dysthymia according to the DSM-5 (300.4).
4. Total HAMD-17 total score of >16 at Screening.
5. Use of mono-aminergic antidepressant drug (SSRI, SNRI, mirtazapine, TCA, MAO-I) at a stable dose for at least 4 weeks (6 weeks for fluoxetine).
6. Must read and speak Dutch as first language and English as second language on secondary school diploma level.
7. Able to comply with the study procedures, prohibitions and restrictions (drug and alcohol use) as specified in the protocol.
8. Android-based smartphone.

Patient Exclusion Criteria

Patients fulfilling **any** of the following criteria are not eligible for inclusion in this study. No additional exclusions may be applied by the investigator, in order to ensure that the study population will be representative of all eligible patients.

1. Current primary DSM-5 diagnosis of general anxiety disorder (GAD), panic disorder, obsessive compulsive disorder (OCD), posttraumatic stress disorder (PTSD), anorexia nervosa, bulimia nervosa or cluster C personality disorder (e.g. avoidant, dependent, obsessive-compulsive personality disorders). Subjects for whom the diagnosed mood disorder (MDD, PDD or dysthymia) is considered the primary diagnosis are not excluded.
2. Current or previous diagnosed psychotic disorder, mood disorder with psychotic features, bipolar disorder, mental retardation, cluster B personality disorder (e.g., borderline, antisocial, narcissistic personality disorders).
3. Current, or recent history of, clinically significant suicidal thoughts or ideation within the past 12 months or any suicidal behavior within the past 6 months as demonstrated with the C-SSRS should be carefully screened and only included at the discretion of the investigator.
4. Positive urine test for drugs of abuse at Screening or on study days or a current diagnosis of substance use disorder (including alcohol but excluding nicotine) or previous substance use disorder (including alcohol but excluding nicotine) within the past 12 months according to DSM-5.
5. Evidence of renal, hepatic, cardiovascular or metabolic dysfunction or any active or chronic disease or condition that could interfere with the conduct of the study, or that would pose an unacceptable risk to the subject in the opinion of the investigator (following a detailed medical

history, physical examination, vital signs (systolic and diastolic blood pressure, pulse rate, body temperature) and 12-lead electrocardiogram (ECG)). Minor deviations from the normal range may be accepted, if judged by the investigator to have no clinical relevance.

6. Clinically significant abnormalities, as judged by the investigator, in laboratory test results (including hepatic and renal panels, complete blood count, chemistry panel and urinalysis). In the case of uncertain or questionable results, tests performed during Screening may be repeated before inclusion to confirm eligibility or to assist in evaluating clinical relevance.
7. Positive urine β-human chorionic gonadotropin (β-hCG) pregnancy test at Screening in women of childbearing potential.
8. Current enrollment in an interventional study.

General Restrictions

Restrictions for all trial participants are drug and alcohol use 48 hours prior to Visit 1 and up to Visit 3.

Assessment Schedule

Table 1.1: Overall assessment schedule

Assessments					
Visit		***Screening***	***Visit 1***	***Visit 2***	***Visit 3***
**Day**		**Day -21 – Day -2**	**Day 1**	**Day 7**	**Day 14**
Informed Consent		X			
Demographics		X			
Inclusion and Exclusion criteria		X			
Medical and psychiatric history		X			
Physical examination, weight/height and vital signs (pulse rate, BP, RR, body temperature)		X			
ECG		X			
Concomitant medication		X	X	X	X
Hematology, Chemistry, Urinalysis, Virology		X			
Pregnancy test (urine β-hCG)		X			
Urine Drug Screening/ Alcohol breath test		X	X	X	X
Soluble biomarkers			X		
Optional DNA Blood Collection			X[1]		
Psychometry	MINI[a], SIGHD	X			
	C-SSRS	X	X	X	X
	MADRS-SIGMA,		X	X	X
Installation/ deinstallation of Sonde, CognitionKit, and BeHapp			X		X
Sonde voice samples			X*	X	X
NeuroCart			X*	X	X
Neurotrack			X*	X	X
ElMindA BNA			X*	X	X
EBT			X*	X	X
Sonde[b]			←twice per week at home →		
CogKit App[c]			←daily at home→		
BeHapp[d]			←Subject continuous at home (passive mode)→		
Subject feedback questionnaire					X
(S)AE recording		X	X	X	X

BP = Blood Pressure, RR = Respiratory Rate, MINI = Mini International Neuropsychiatric Interview, SIGH-D = Structured Interview Guide for the Hamilton Depression Rating Scale, MADRS = Montgomery–Åsberg Depression Rating Scale, SIGMA = structured interview guide for the MADRS, C-SSRS = Columbia-Suicide Severity Rating Scale, BNA = Brain Network Activation, (S)AE = Serious Adverse Event, EEG = Electroencephalogram

[1]DNA sampling was optional and if subjects participated, they must have provided a separate signature.

[a] only for MDD patients

[b] subjects continued participating in the voice samples on the mobile phones or devices by reading passages aloud, freely speaking, or performing simple cognitive tasks.

[c] daily assessment of mood and cognition using an Android device to measure two questions adapted from the PHQ-9 and the 2-Back.

[d] BeHapp was a behavioural monitoring service for the collection and analyses of smartphone based behavioural data.

* training only

Table 1.2: Detailed assessment schedule at Visit 2 and Visit 3

Assessments	Admission	60min	100min	160min	190 min	230min	270min	290
C-SSRS, MADRS-SIGMA	X							
Sonde voice samples[a]	X							
NeuroCart[b]		X		X		X		
ElMindA BNA[c]			X					
Neurotrack[d]					X			
EBT							X	
Discharge from Clinic								X

C-SSRS = Columbia-Suicide Severity Rating Scale

MADRS = Montgomery–Åsberg Depression Rating Scale, SIGMA = structured interview guide for the MADRS

[a] in-clinic voice samples in addition to continuous monitoring at-home with a personal or assigned mobile device. The tests include a sentence/passage reading, free response, a Stroop task and a questionnaire on caffeine and alcohol use. Passively the device will record ambient sound

[b] body sway, smooth pursuit eye movements, pupil size, saccadic peak velocity, adaptive tracking, Stroop test, N-back and Bond & Lader and Bowdle Visual Analogue Scales

[c] auditory Oddball task, Visual Go-No-Go and Resting EEG eyes-closed

[d] tracking eye movements during Visual Paired Comparison, Paired Associates Learning & Recognition Memory and Self-report Questionnaire

Patient Characteristics

Figure 1.1: Duration of the antidepressant medication for the study patients

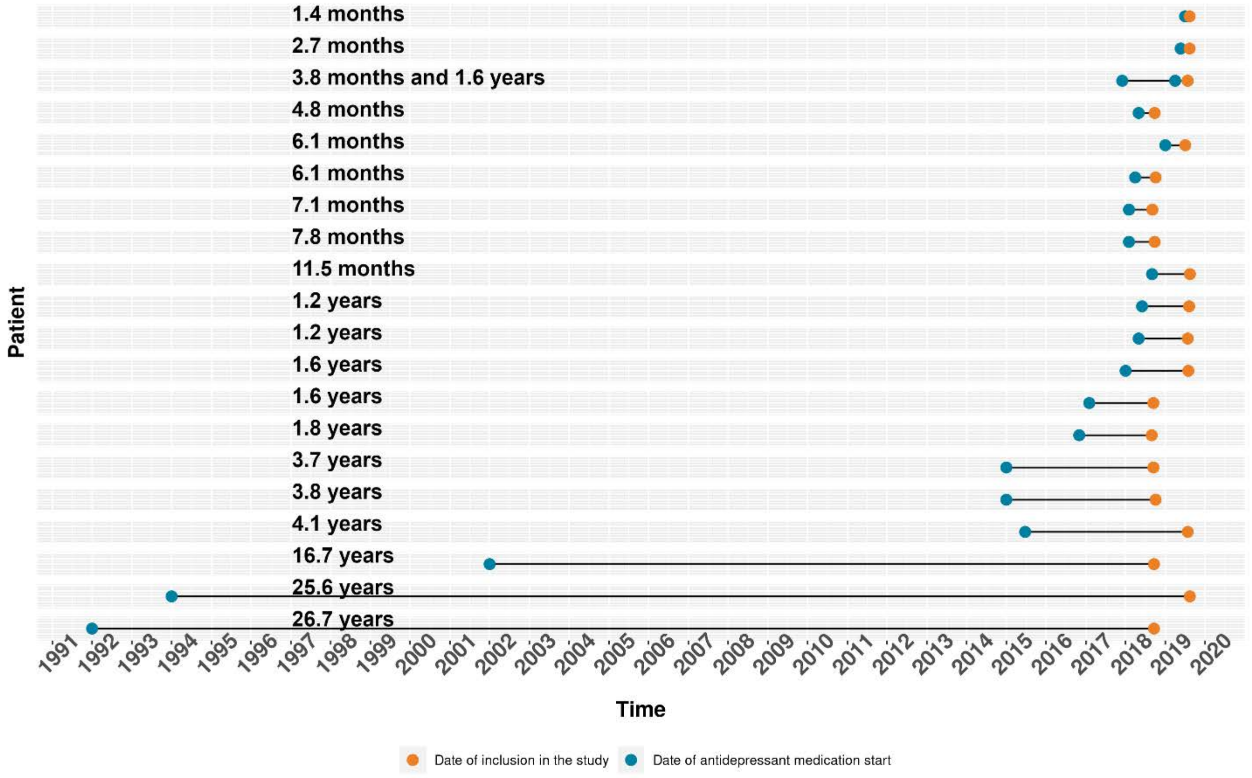

Cognition Kit

The CognitionKit mobile app was used to assess cognition and mood. Longitudinal data per subject was acquired, i.e. the parameters were calculated each time the subject engaged with the app, on a daily basis. The number of measurements per subject could vary. Overall, 38 subjects (19 unipolar depression and 19 healthy) had valid Cognition Kit data in the analysis dataset.

- **Cognitive assessment** was based on the 2-back working memory test. For this test, nine symbols (randomly selected from a pool of 227) are presented for 1 second one at a time over 30 trials. Subjects are asked to make a response when any symbol is the same as the one presented 2 trials earlier. The outcome measure was dPrime, defined as the ratio of hits (correct detection of a 2-Back match) to false alarms (response during no match).
- **Mood assessment** involved two questions related to depressed mood, asked using a chat bot. A response to each question was scored from 0 to 3, with 3 representing the greatest severity of the symptom. A total score, PHQ2, was obtained as the sum of two responses on a scale 0–6.

Figure 2.1 shows individual profiles and mean (SD) of PHQ2 over time. There is a clear separation between healthy and depressed subjects.

Figure 2.2 shows individual profiles and mean (SD) of dPrime over time. There is a high overlap between healthy and depressed subjects. Also, there is some evidence of a learning effect – an increasing trend in 2-Back over time.

For correlation and regression analyses, for each subject we derived average values of PHQ2 and dPrime by averaging all available valid observations within subject.

Figure 2.3 shows pairwise correlations of PHQ2, 2-Back, and MADRS. MADRS total score had strong positive correlation with PHQ2 (r = 0.9).

Table 2.1 shows the results of the LOOCV logistic regression model with PHQ2 as a predictor.

Table 2.2 shows the results of the LOOCV linear regression model of MADRS with PHQ2 (linear and quadratic terms) as a predictor.

Figure 2.1: Individual profiles and mean (SD) of PHQ2 vs. study day

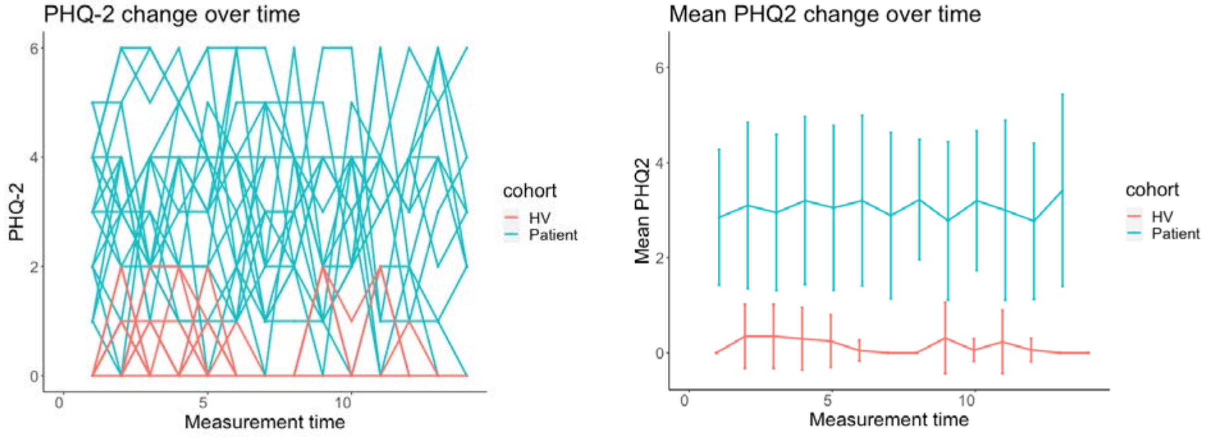

Figure 2.2: Individual profiles and mean (SD) of dPrime vs. study day

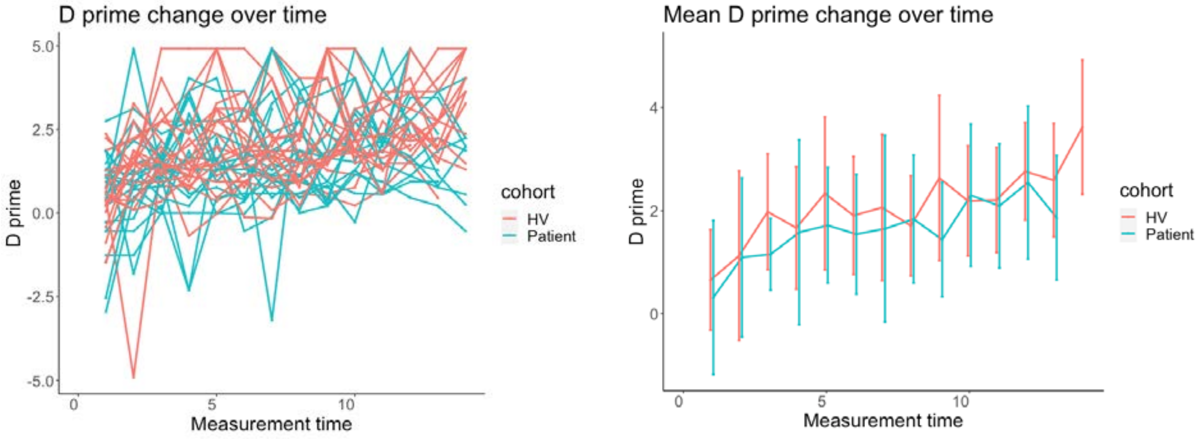

Figure 2.3: Pairwise correlations of Cognition Kit features and MADRS

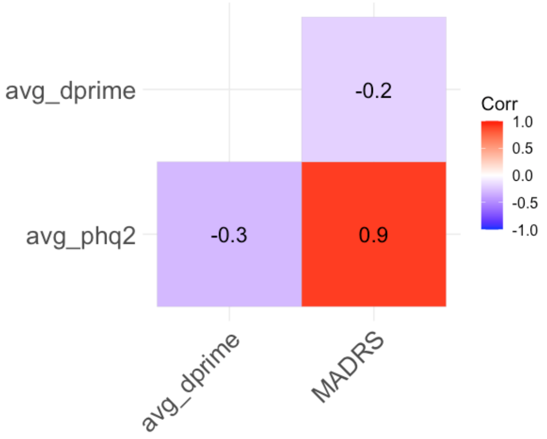

Table 2.1: Cambridge Cognition - Results of a logistic regression analysis

Dataset	Selected variable(s)	Beta (β)	Std. Error	z value	p-value	McFadden Pseudo adjusted R^2
N=38 (n=19 healthy; n=19 unipolar depression	Intercept	-3.711	1.264	-2.936	0.003	0.791
	PHQ2	3.667	1.592	2.303	0.021	

ROC AUC = Area under the receiver operating characteristics (ROC) curve

Table 2.2: Cambridge Cognition - Results of a linear regression analysis of MADRS

Dataset	Selected variables	Beta (β)	Std. Error	t value	p-value	Adj.R^2
N=38 (n=19 healthy; n=19 unipolar depression)	Intercept	-0.171	1.225	-0.140	0.889	0.858
	PHQ2	13.819	1.630	8.475	5.33×10^{-10}	
	PHQ2^2	-1.653	0.379	-4.357	1.10×10^{-4}	

Neurotrack

Data from Neurotrack were ascertained at each in-clinic visit. The procedure can be described as follows: a participant is shown a series of paired images on the laptop screen while the web camera is recording a video of the participant's face. During a familiarization phase, 10 pairs of images are shown. During the test phase, 20 pairs of images are shown, such that one image is always new and the other one was seen earlier in the familiarization phase. The main outcome measures of this test are: (1) *novelty preference*, calculated as percentage of time a participant is viewing the novel image and (2) *oscillation count*, calculated as the number of times a participant is switching from one image to the other. The following features were derived for each participant at a given visit:

- **Mean novelty preference** – average of the 20 novelty performance trials (scale: 0–1).
- Standard deviation across the 20 novelty performance trials (scale: 0–1).
- **Mean oscillation count** – average across oscillation counts of 20 trials.
- **Subjective memory impairment (SMI)** – a self-reported score calculated based on the subjective cognitive report (scale: 1–9).

There were 39 subjects (20 unipolar depression and 19 healthy) with valid Neurotrack data in the analysis dataset.

Figure 3.1 shows mean (SD) of Neurotrack features per visit. There is a clear separation between the groups with respect to SMI, but not any other feature.

Figure 3.2 shows a correlation matrix for Neurotrack features and MADRS. There is a high positive correlation between SMI (avg_mm_ip) and MADRS (r = 0.9).

Table 3.1 shows the results of the LOOCV logistic regression model with SMI as a predictor.

Table 3.2 shows the results of the LOOCV linear regression model of MADRS with SMI as a predictor.

Figure 3.1: Mean (SD) of Neurotrack features vs. visit

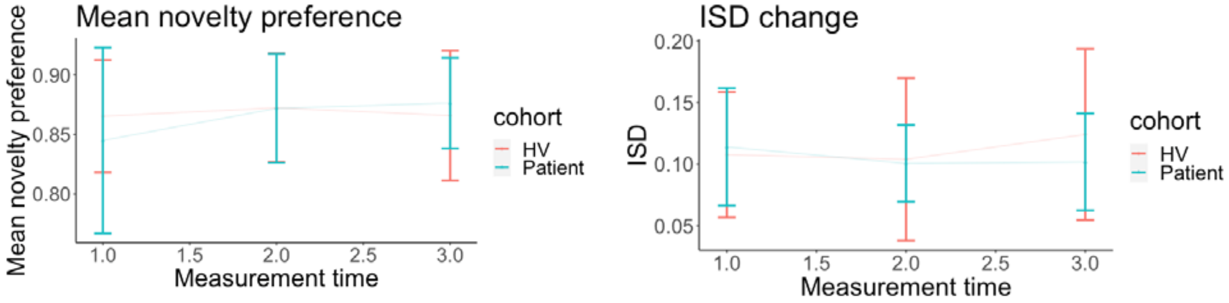

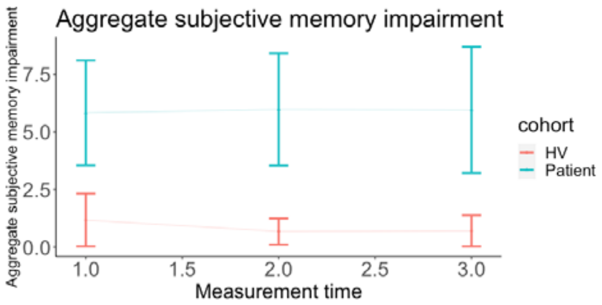

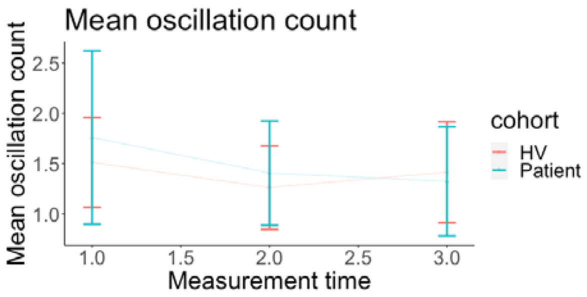

Figure 3.2: Pairwise correlations of Neurotrack features and MADRS

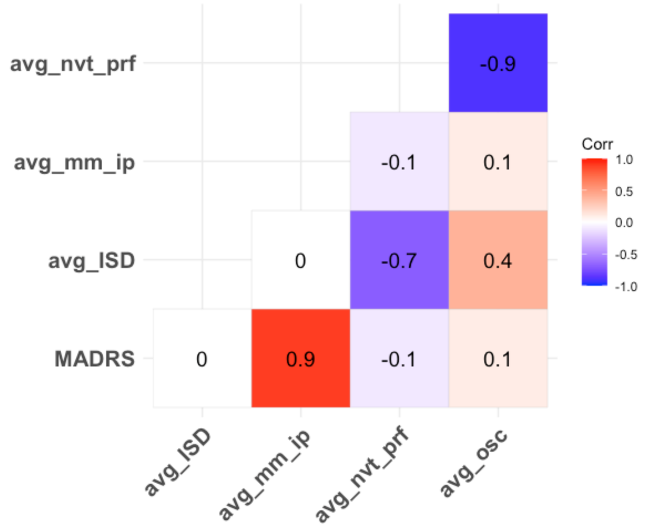

Table 3.1: Neurotrack - Results of a logistic regression analysis

Dataset	Selected variable(s)	Beta (β)	Std. Error	z value	p-value	McFadden Pseudo adjusted R^2
N=39 (n=19 healthy; n=20 unipolar depression	Intercept	-3.387	1.035	-3.272	0.001	0.655
	SMI	1.360	0.473	2.876	0.004	

Table 2.2: Neurotrack - Results of a linear regression analysis of MADRS

Dataset	Selected variables	Beta (β)	Std. Error	t value	p-value	Adj.R^2
N=39 (n=19 healthy; n=20 unipolar depression)	Intercept	0.979	1.654	0.592	0.558	0.737
	SMI	3.713	0.359	10.358	1.75×10^{-12}	

Neurocart

Neurocart was conducted at all 3 visits. Data from visits 2 and 3 were included for analysis as visit 1 was for training purpose. The technology tests were administered three times—at 60, 160, and 230 minutes after admission. A total of 43 features were derived per subject per visit (at any visit, average values across the 3 time point assessments were calculated). These features can be classified into six test categories:

Test category	Description	Variable(s)
Adaptive tracking test	Measures visuomotor coordination and attention	average tracking performance (%)
N-back test	Measures working memory	• average reaction time (msec) for zero/one/two-back tests<br>• [#correct words – #incorrect words] / [total #words] for zero/one/two-back tests
Saccadic eye movements	Measures sedation	• saccadic peak velocity (degrees/second)<br>• saccadic reaction time (seconds)<br>• saccadic inaccuracy (%)
Smooth pursuit eye movements and Body Sway	Measures motor coordination	• average percentage of smooth pursuit (%)<br>• body sway (mm)
Pupillometry	Measures autonomic nervous system activity	• left pupil/iris ratio<br>• right pupil/iris ratio
Visual Analogue Scale (VAS) VAS scales	Subjective measures	16 VAS assessments (scale: 0–100)
Bowdle VAS scales	Subjective measures	13 VAS assessments (scale: 0–100)

All 40 subjects (20 unipolar depression and 20 healthy) had valid Neurocart data and were included in the analysis.

Figure 4.1 shows a matrix of pairwise correlations among Neurocart features and MADRS. Some features (e.g. different visual analogue scale (VAS) assessments) are highly correlated, and reduction of data dimensionality may be useful. Also, MADRS has a moderate correlation with some, but not all, VAS assessments.

Table 4.1 shows the results of the LOOCV logistic regression model with Neurocart features as predictors.

Table 4.2 shows the results of the LOOCV linear regression model of MADRS with Neurocart features as predictors.

Figure 4.1: Pairwise correlations of Neurocart features and MADRS

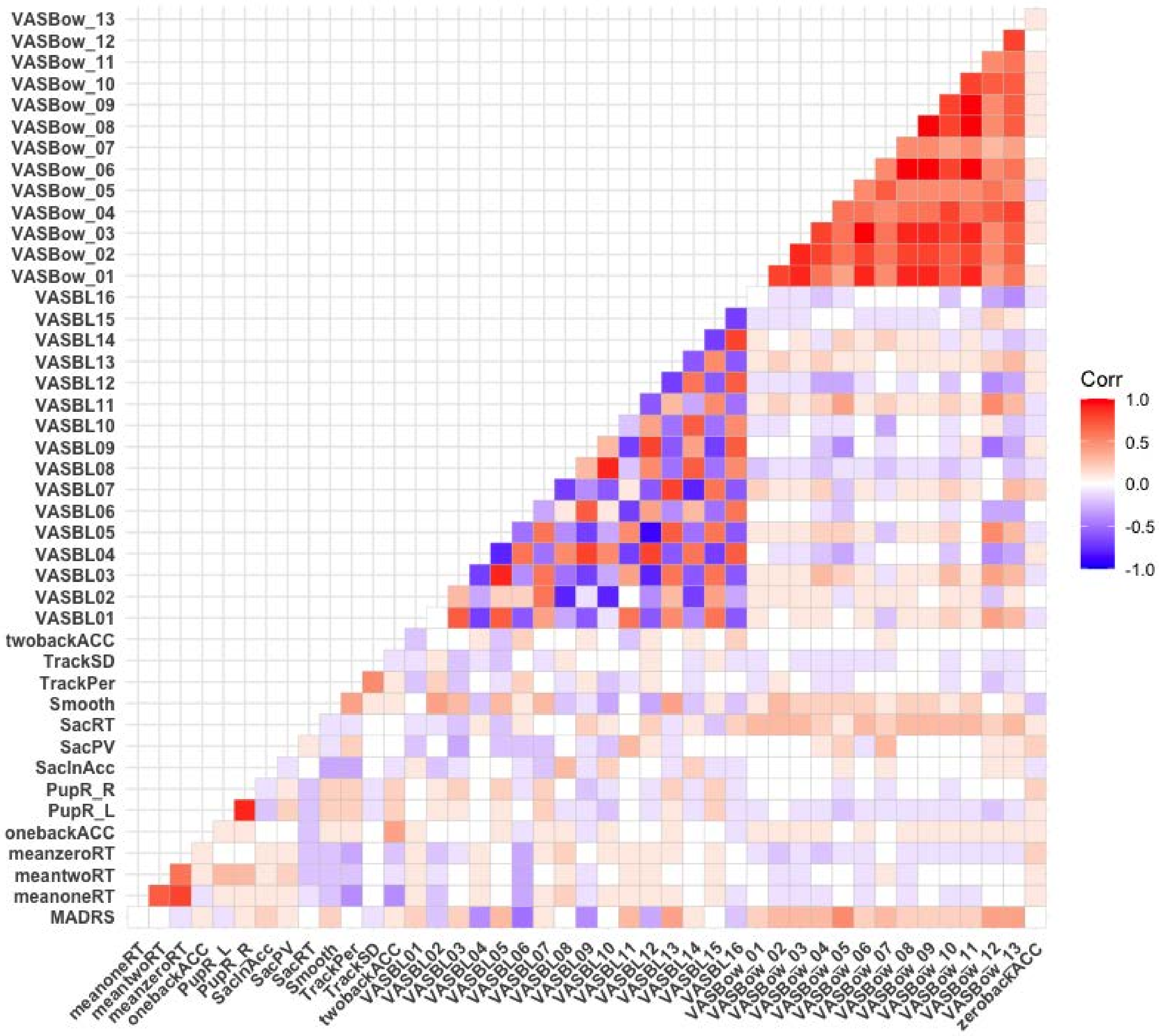

Table 4.1: Neurocart - Results of a logistic regression analysis

Dataset	Selected variable(s)	Beta (β)	Std. Error	z value	p-value	McFadden Pseudo adjusted R^2
N=40 (n=20 healthy; n=20 unipolar depression	Intercept	-6.609	11.102	-0.595	0.552	0.425
	twobackACC	33.638	15.321	2.196	0.028	
	VASBL06	-0.347	0.113	-3.084	2.04×10^{-3}	
	VASBL15	-0.210	0.084	-2.491	0.012	

Table 4.2: Neurocart - Results of a linear regression analysis of MADRS

Dataset	Selected variables	Beta (β)	Std. Error	t value	p-value	Adj.R^2
N=40 (n=20 healthy; n=20 unipolar depression)	Intercept	-8.497	23.774	-0.357	0.723	0.561
	twobackACC	59.098	25.854	2.286	0.029	
	VASBL06	-0.622	0.138	-4.524	7.45×10^{-5}	
	VASBL13	0.329	0.127	2.596	0.014	
	VASBL15	-0.476	0.167	-2.848	0.008	
	VASBow_01	-2.454	0.840	-2.921	0.006	
	VASBow_03	3.513	1.100	3.195	0.003	

BeHapp

The BeHapp application passively accessed various sources of data on the phone, including communication events, phone usage logs, geographic location data, and Wi-Fi sensor data. Multiple features were extracted per subject. The following 10 features, from two categories (Location and Communication) were considered in the analysis:

Data category	Variable name	Description
Location	BeHapp1	Total number of stay points
Location	BeHapp2	Number of places visited once
Location	BeHapp3	Number of unique places visited
Location	BeHapp4	Total amount of time spent at home in minutes
Location	BeHapp5	Average distance from home
Communication	BeHapp6	Average repetition per contact for outgoing, incoming and missed calls
Communication	BeHapp7	Total number of whatsapp calls
Communication	BeHapp8	Entropy of the usage time of communication apps
Communication	BeHapp9	Mean usage time of communication apps
Communication	BeHapp10	Total count of communication apps usage

There were 30 subjects (16 unipolar depression and 14 healthy) with valid Neurotrack data in the analysis dataset.

Figure 5.1 shows a matrix of pairwise correlations among BeHapp features and MADRS. Some features (e.g. BeHapp2 vs. BeHapp3; BeHapp7 vs. BeHapp10) are highly correlated, and reduction of data dimensionality may be useful. Also, MADRS has moderate negative correlation with BeHapp7 (r = -0.42), BeHapp8 (r = -0.31) and BeHapp10 (r = -0.42). Therefore, one may conjecture that greater values of communication activity are associated with lower depressive symptoms.

Table 5.1 shows the results of the LOOCV logistic regression model with BeHapp features as predictors.

Table 5.2 shows the results of the LOOCV linear regression model of MADRS with BeHapp features as predictors.

Figure 5.1: Pairwise correlations of BeHapp features and MADRS

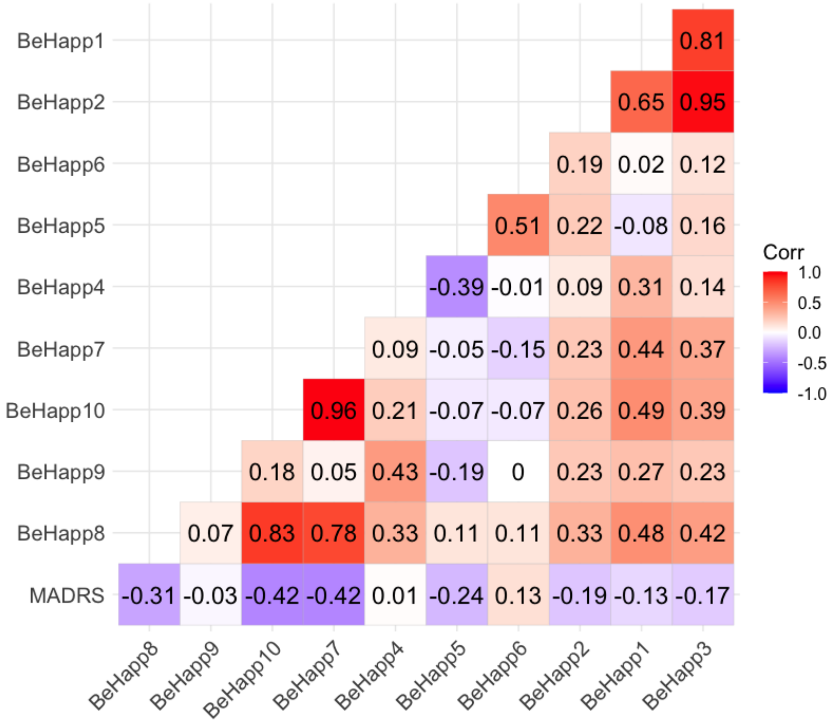

Table 5.1: BeHapp - Results of a logistic regression analysis

Dataset	Selected variable(s)	Beta (β)	Std. Error	z value	p-value	McFadden Pseudo adjusted R^2
N=28 (n=14 healthy; n=14 unipolar depression)	Intercept	1.836	0.868	2.116	0.034	0.150
	BeHapp10	-0.002	0.001	-2.382	0.017	

Table 5.2: BeHapp - Results of a linear regression analysis of MADRS

Dataset	Selected variables	Beta (β)	Std. Error	t value	p-value	Adj.R^2
N=28 (n=14 healthy; n=14 unipolar depression)	Intercept	10.337	7.057	1.465	0.157	0.2745
	BeHapp2	-2.287	1.329	-1.722	0.099	
	BeHapp3	1.950	1.136	1.716	0.100	
	BeHapp5	0	0	-2.160	0.042	
	BeHapp6	2.927	1.51	1.939	0.065	
	BeHapp10	-0.014	0.005	-2.957	0.007	

ElMindA

ElMindA's EEG-based brain network analysis platform was applied at three in-clinic visits. At each visit, a total of 47 features were derived per subject. These features can be classified into two major categories:

- Resting state EEG: 27 features (9 features from each of the alpha, beta, and gamma power spectrum).
- BNA: 20 BNA features from the auditory oddball (AOB) task and visual go-no-go (VGNG) task:

	Feature	Description
1	BNA_ACCURACY	BNA - ACCURACY
2	BNA_ADIA	BNA - ATTENTION DRIVEN INHIBITION (ATTENTION TO MEMORY LINK) AMPLITUDE
3	BNA_ADIL	BNA - ATTENTION DRIVEN INHIBITION (ATTENTION TO MEMORY LINK) LATENCY
4	BNA_APA	BNA - AUDITORY PROCESSING (SUSTAINED ATTENTION) AMPLITUDE
5	BNA_APL	BNA - AUDITORY PROCESSING (SUSTAINED ATTENTION) LATENCY
6	BNA_EAPL	BNA - EARLY AUDITORY PROCESSING LATENCY
7	BNA_FA_AOB	BNA - FILTERING OF INFORMATION (AOB) AMPLITUDE
8	BNA_FA_VGNG	BNA - FILTERING OF INFORMATION (VGNG) AMPLITUDE
9	BNA_FIA	BNA - FILTERING OF INFORMATION (TARGET) AMPLITUDE
10	BNA_FL_AOB	BNA - FILTERING OF INFORMATION (AOB) LATENCY
11	BNA_FL_VGNG	BNA - FILTERING OF INFORMATION (VGNG) LATENCY
12	BNA_FT_AOB	BNA - FILTERING OF INFORMATION (AOB) TOPOGRAPHY
13	BNA_ICA	BNA - INHIBITORY CONTROL AMPLITUDE
14	BNA_ICL	BNA - INHIBITORY CONTROL LATENCY
15	BNA_MIA	BNA - MOTOR INHIBITION AMPLITUDE
16	BNA_MIL	BNA - MOTOR INHIBITION LATENCY
17	BNA_NCF	BNA - NEURAL CONSISTENCY FREQUENT
18	BNA_NCN	BNA - NEURAL CONSISTENCY NOVEL
19	BNA_NCT	BNA - NEURAL CONSISTENCY TARGET
20	BNA_RT	BNA - REACTION TIME (AOB)

All 40 subjects (20 unipolar depression and 20 healthy) had valid resting stage EEG data and were included in the EEG analysis.

38 subjects (20 unipolar depression and 18 healthy) had valid BNA data and were included in the BNA analysis.

Figure 6.1 shows a matrix of pairwise correlations among 27 resting state EEG features, and a similar matrix for 20 BNA features. Within each matrix, we also have the total MADRS score (bottom row). It can be seen that:

- For the resting state EEG data, variables within each of the alpha and beta spectrum are highly positively correlated (r = 0.8 to 1.0). Also, there is moderate negative correlation (r = -0.4 to -0.5) between variables in the alpha power and gamma power spectra. Reduction of data dimensionality may be useful by removing some of the highly correlated features.
- MADRS has moderate negative correlation (r = -0.3 to -0.4) with variables in the alpha power spectrum.
- MADRS has relatively low correlation with BNA features.

Table 6.1 shows the results of the LOOCV logistic regression models with EEG and BNA features as predictors.

Table 6.2 shows the results of the LOOCV linear regression models of MADRS with EEG and BNA features as predictors.

Figure 6.1: Pairwise correlations of ElMindA features and MADRS

(a) Resting state EEG features

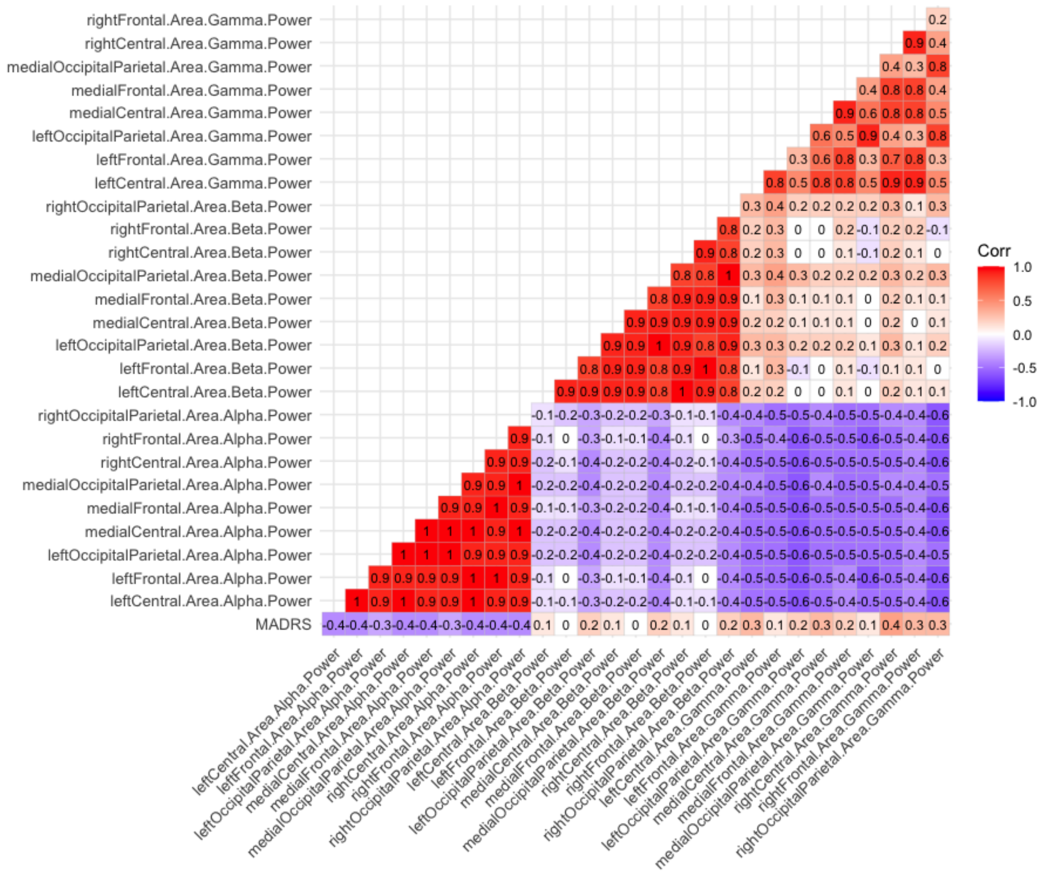

(b) BNA features

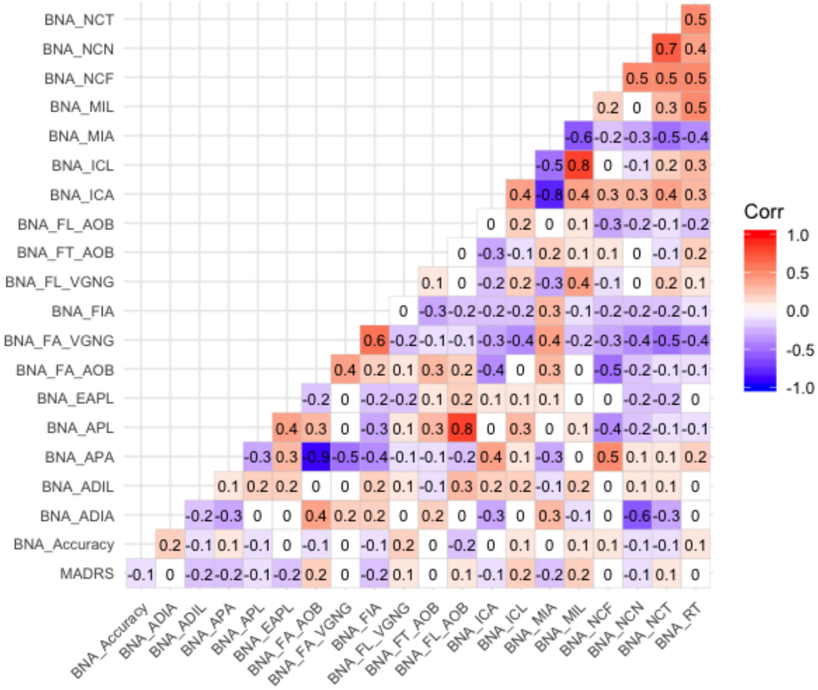

Table 6.1: ElMindA - Results of a logistic regression analysis

(a) Resting state EEG features

Dataset	Selected variable(s)	Beta (β)	Std. Error	z value	p-value	McFadden Pseudo adjusted R^2
N=40 (n=20 healthy; n=20 unipolar depression	Intercept	5.051	3.305	1.528	0.127	0.303
	rightCentral.Area.Alpha.Power	-0.120	0.066	-1.813	0.070	
	rightFrontal.Area.Beta.Power	-0.783	0.435	-1.801	0.072	
	rightOccipitalParietal.Area.Beta.Power	0.639	0.358	1.785	0.074	
	leftFrontal.Area.Gamma.Power	-1.998	0.894	-2.235	0.025	
	medialOccipitalParietal.Area.Gamma.Power	-1.609	0.813	-1.978	0.048	
	rightCentral.Area.Gamma.Power	1.873	0.778	2.408	0.016	

(b) BNA features

Dataset	Selected variable(s)	Beta (β)	Std. Error	z value	p-value	McFadden Pseudo adjusted R^2
N=38 (n=18 healthy; n=20 unipolar depression	Intercept	0.515	0.912	0.565	0.572	0.012
	BNA_ICA	-0.357	0.206	-1.732	0.083	
	BNA_MIA	-0.549	0.289	-1.896	0.058	

Table 6.2: ElMindA - Results of a linear regression analysis of MADRS

(a) Resting state EEG features

Dataset	Selected variables	Beta (β)	Std. Error	t value	p-value	Adj.R^2
N=40 (n=20 healthy; n=20 unipolar depression)	Intercept	20.994	7.602	2.762	0.009	0.347
	leftCentral.Area.Alpha.Power	-0.369	0.154	-2.387	0.023	
	leftCentral.Area.Beta.Power	-6.545	2.367	-2.765	0.009	
	rightCentral.Area.Beta.Power	6.670	2.340	2.851	0.007	
	leftCentral.Area.Gamma.Power	4.347	1.424	3.053	0.004	
	leftFrontal.Area.Gamma.Power	-4.731	1.529	-3.094	0.004	

(b) BNA features

Dataset	Selected variables	Beta (β)	Std. Error	t value	p-value	Adj.R^2
N=38 (n=18 healthy; n=20 unipolar depression	Intercept	41.296	40.721	1.014	0.319	0.212
	BNA_Accuracy	-0.339	0.180	-1.880	0.07	
	BNA_APL	-0.581	0.294	-1.978	0.057	
	BNA_FA_VGNG	3.862	1.907	2.025	0.052	
	BNA_FIA	-3.698	1.685	-2.195	0.036	
	BNA_ICA	-2.75	1.018	-2.701	0.011	
	BNA_ICL	0.192	0.105	1.821	0.079	
	BNA_MIA	-3.607	1.456	-2.478	0.019	

Sonde Health

Sonde data were acquired through the Sonde Health smartphone app. The raw data (voice samples) were processed using Sonde's proprietary algorithms to extract a total of 72 Sonde variables per subject. These variables can be grouped into nine categories based on the test from which they were calculated:

1. SONDE_TEST1 (BASELINE–AHHH): 8 parameters (SONDE0, SONDE1, SONDE2, SONDE3, SONDE4, SONDE5, SONDE6, SONDE7).
2. SONDE_TEST2 (BASELINE–PA_TA_KA): 8 parameters (SONDE9, SONDE10, SONDE11, SONDE12, SONDE13, SONDE14, SONDE15, SONDE16).
3. SONDE_TEST3 (BASELINE–SHORT_READING): 8 parameters (SONDE17, SONDE18, SONDE19, SONDE20, SONDE21, SONDE22, SONDE23, SONDE24).
4. SONDE_TEST4 (FOCUS–STROOP): 8 parameters (SONDE25, SONDE26, SONDE27, SONDE28, SONDE29, SONDE30, SONDE31, SONDE32).
5. SONDE_TEST5 (FOCUS–STROOP): 8 parameters (SONDE33, SONDE34, SONDE35, SONDE36, SONDE37, SONDE38, SONDE39, SONDE40).
6. SONDE_TEST6 (FOCUS–STROOP): 8 parameters (SONDE41, SONDE42, SONDE43, SONDE44, SONDE45, SONDE46, SONDE47, SONDE48).
7. SONDE_TEST7 (FOCUS–STROOP): 8 parameters (SONDE49, SONDE50, SONDE51, SONDE52, SONDE53, SONDE54, SONDE55, SONDE56).
8. SONDE_TEST8 (FREE_SPEECH): 8 parameters (SONDE57, SONDE58, SONDE59, SONDE60, SONDE61, SONDE62, SONDE63, SONDE64).
9. SONDE_TEST4-7 (FOCUS–STROOP): 8 parameters (FOCUS0, FOCUS1, FOCUS2, FOCUS3, FOCUS4, FOCUS5, FOCUS6, FOCUS7).

All 40 subjects (20 unipolar depression and 20 healthy) had valid Sonde data and were included in the analysis.

Figure 7.1 shows a matrix of pairwise correlations among Sonde features and MADRS. It is difficult to see any clear pattern. From the bottom row of the matrix, MADRS had overall low correlation with Sonde features; the most notable negative correlation value was -0.4.

Table 7.1 shows the results of the LOOCV logistic regression models with Sonde features as predictors.

Table 7.2 shows the results of the LOOCV linear regression models of MADRS with Sonde features as predictors.

Figure 7.1: Pairwise correlations of Sonde features and MADRS

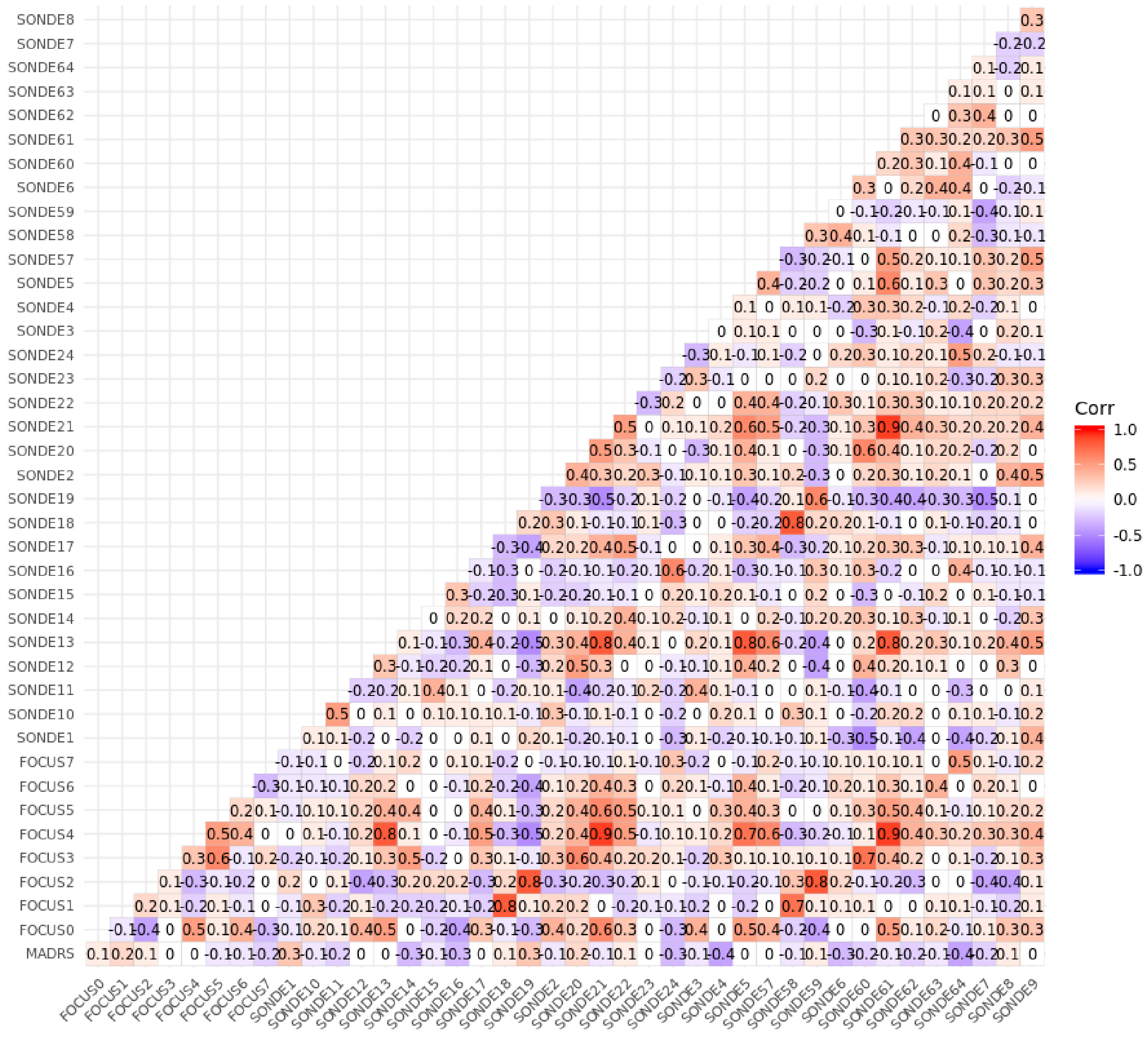

Table 7.1: Sonde - Results of a logistic regression analysis

Dataset	Selected variable(s)	Beta (β)	Std. Error	z value	p-value	McFadden Pseudo adjusted R^2
N=40 (n=20 healthy; n=20 unipolar depression)	Intercept	-9.560	4.603	-2.077	0.038	0.158
	FOCUS2	-0.091	0.046	-1.989	0.047	
	FOCUS3	-0.946	0.450	-2.101	0.036	
	SONDE19	0.120	0.046	2.609	0.009	
	SONDE20	1.433	0.528	2.716	0.007	

Table 7.2: Sonde - Results of a linear regression analysis of MADRS

Dataset	Selected variables	Beta (β)	Std. Error	t value	p-value	Adj.R^2
N=40 (n=20 healthy; n=20 unipolar depression)	Intercept	-32.688	20.973	-1.559	0.128	0.226
	FOCUS2	-0.202	0.167	-1.214	0.233	
	FOCUS3	-2.913	1.610	-1.809	0.079	
	SONDE19	0.406	0.133	3.056	0.004	
	SONDE20	4.829	1.580	3.056	0.004	

Emotional Bias Task (EBT)

The EBT was administered for a sub-sample of 20 participants (10 unipolar depression and 10 healthy) at three time points, one week apart (baseline, day 7 and day 14). The key outcome measure was *bias point*, which represents the number of trials on which “happy” was chosen as the label for the ambiguous facial expression the participants were presented with. For instance, a bias point of 15 would indicate always selecting “happy” whereas a bias point of 7.5 indicates zero bias. The EBT features are described below.

Data category	Feature name	Description
Sad trials	EBTRTSDS	EBT STANDARD DEVIATION REACTION TIME SAD (MS)
Sad trials	EBTRTMNS	EBT MEAN REACTION TIME SAD (MS)
Sad trials	EBTRTMDS	EBT MEDIAN REACTION TIME SAD (MS)
Sad trials	EBTRCS	EBT RESPONSE COUNT SAD
Happy trials	EBTRTSDH	EBT STANDARD DEVIATION REACTION TIME HAPPY (MS)
Happy trials	EBTRTMNH	EBT MEAN REACTION TIME HAPPY (MS)
Happy trials	EBTRTMDH	EBT MEDIAN REACTION TIME HAPPY (MS)
Happy trials	EBTRCH	EBT RESPONSE COUNT HAPPY
All trials	EBTRTSDT	EBT STANDARD DEVIATION REACTION TIME TOTAL (MS)
All trials	EBTRTMNT	EBT MEAN REACTION TIME TOTAL (MS)
All trials	EBTRTMDT	EBT MEDIAN REACTION TIME TOTAL (MS)
All trials	EBTBP	EBT BIAS POINT

Figure 8.1 shows a matrix of pairwise correlations among EBT features and MADRS. Some features of the EBT were highly correlated, indicating that reduction of data dimensionality may be useful. MADRS had moderate negative correlation with EBT bias point (r = -0.4) and EBT response count Happy (r = -0.4), and moderate positive correlation with EBT response count Sad (r = 0.4).

Table 8.1 shows the results of the LOOCV logistic regression models with bias point as predictor.

Table 8.2 shows the results of the LOOCV linear regression models of MADRS with bias point as predictor.

Figure 8.1: Pairwise correlations of EBT features and MADRS

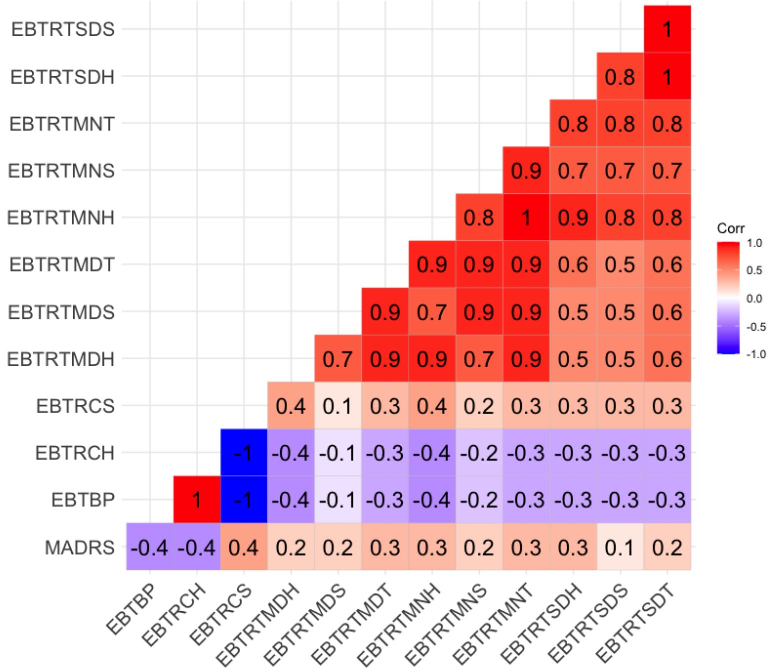

Table 8.1: EBT - Results of a logistic regression analysis

Dataset	Selected variable(s)	Beta (β)	Std. Error	z value	p-value	McFadden Pseudo adjusted R^2
N=20 (n=10 healthy; n=10 unipolar depression)	Intercept	3.794	2.512	1.511	0.131	0.025
	EBTBP	-0.511	0.334	-1.530	0.126	

Table 8.2: EBT - Results of a linear regression analysis of MADRS

Dataset	Selected variables	Beta (β)	Std. Error	t value	p-value	Adj.R^2
N=20 (n=10 healthy; n=10 unipolar depression)	Intercept	38.169	14.210	2.686	0.015	0.107
	EBTBP	-3.387	1.869	-1.812	0.086	
